# Mapping intron retention events contributing to complex traits using splice quantitative trait locus

**DOI:** 10.1186/s13007-023-01048-4

**Published:** 2023-07-21

**Authors:** Siyuan Wang, Hongyu Wu, Yongyan Zhao, Luyao Wang, Xueying Guan, Ting Zhao

**Affiliations:** 1 Zhejiang Provincial Key Laboratory of Crop Genetic Resources, Institute of Crop Science, Plant Precision Breeding Academy, College of Agriculture and Biotechnology, Zhejiang University, Hangzhou, 300058 China; 2 Hainan Institute of Zhejiang University, Building 11, Yonyou Industrial Park, Yazhou Bay Science and Technology City, Yazhou District, Sanya, 572025 Hainan China

**Keywords:** Alternative splicing (AS), Intron retention (IR), Cotton (*Gossypium hirsutum* L.), Fiber yield, Splicing quantitative trait locus (sQTL)

## Abstract

**Background:**

Alternative splicing (AS) of mRNA plays an important roles in transcriptome diversity, involving regulation of plant growth and stress response. Understanding the variation of AS events underlying GWAS loci in a crop population can provide insight into the molecular mechanisms of complex agronomic traits. To date, genome-wide association studies relating AS events to agronomic traits have rarely been conducted at the population level in crops.

**Results:**

Here, a pipeline was constructed to identify candidate AS events related to complex traits. Firstly, ovule transcriptome data were used to characterize intron retention (IR), the predominant type of AS in plants, on a genome-wide scale. This was done in a natural population consisting of 279 upland cotton lines. Secondly, splice quantitative trait locus (sQTL) analysis was carried out, which yielded a total of 2295 sQTLs involving 1607 genes. Of these, 14.25% (*n* = 427) were *cis*-regulatory loci. Integration with expression quantitative trait loci (eQTL) revealed that 53 (21.4%) *cis*-sGenes were regulated by both *cis*-sQTLs and *cis*-eQTLs. Finally, co-localization analysis integrated with GWAS loci in this population showed 32 *cis*-QTLs to be co-located with genetic regulatory loci related to fiber yield and quality traits, indicating that sQTLs are likely to participate in regulating cotton fiber yield and quality. An in-depth evaluation confirmed that differences in the IR rates of sQTL-regulated candidate genes such as *GhLRRK1* and *GhGC1* are associated with lint percentage (LP), which has potential in breeding applications.

**Conclusion:**

This study provides a clue that AS of mRNA has an impact on crop yield, along with functional sQTLs are new genetic resources for cotton precision breeding.

**Supplementary Information:**

The online version contains supplementary material available at 10.1186/s13007-023-01048-4.

## Background

Cotton is one of the most important sources of natural fiber and cash crops worldwide [1]. Allotetraploid upland cotton (AD)_1_ (*Gossypium hirsutum* L.) accounts for more than 90% of cultivated cotton and is the main source of renewable textile fiber [2]. The global textile industry has a continuous and stable consumer demand for cotton fiber, and increasing yield has long been an important goal of cotton breeding. However, cotton yield traits are complex quantitative traits controlled by polygenes and affected by environmental conditions. The mechanisms of genetic impacts on complex quantitative traits include but are not limited to gene structural variations and associated effects on alternative splicing, amino acid coding, and so on.

With the development of high-throughput sequencing technology, molecular markers have become widely used in determination genetic loci that influence cotton yield traits [3–6]. Genome-wide association studies (GWAS) represent an effective method for locating genetic factors that underpin complex traits at a genome-wide scale. This association study can analyze the correlations between single nucleotide polymorphism (SNP) markers and noted phenotypes to identify candidate genomic regions that may impact a phenotype. GWAS was first successfully implemented in upland cotton for the identification of SNP loci and candidate loci for fiber quality and yield traits using a China upland cotton population (CUCP1) collected from three representative ecological cotton-producing regions of China: the Yellow River, Yangtze River, and Xinjiang cotton-growing areas [7]. However, GWAS signals are localized to regions with genetic structural variation, and their resolution is limited by population structure and size; moreover, the majority of the GWAS often do not identify a specific gene that has a deterministic effect on phenotype, due to limitations in sample size and marker number. Genetic structural variation affects the phenotype through multiple aspects, including alternations on transcription and post transcription levels. Expression quantitative trait locus (eQTL) studies have increasingly been integrated with GWAS loci to improve the accuracy and interpret those functional variants with potential biological mechanisms [8, 9].

In addition to the modulation of the overall transcription level, gene transcripts may undergo alternative splicing (AS) [10], in which different splice sites produce multiple mRNA variants from a single gene [11, 12]. AS is an important mechanism for controlling gene expression and further increasing proteome complexity. In plants, intron retention (IR) is the most prevalent form of AS [13], accounting for 23–47% of AS events [14–16]. Retention of an intron can result in the alternative mRNA with a possibility of harboring a premature termination codon (PTC). If this PTC is located upstream of an exon–exon junction (more than 50 nucleotides), the mRNA will be degraded by nonsense-mediated decay (NMD) [17]. However, IR isoforms are usually not targets for the NMD pathway [18]. Some mRNAs with PTCs can instead be translated into truncated proteins, which may potentially lack one or more active structural domains of the full-length protein [19]. Some transcripts with retained introns have been shown to serve specific functions in plants, such as in flowering [20, 21] and stress response [19]. In addition, intron-retaining mRNAs can become potential targets of miRNAs to be degraded [22].

With the development and application of sequencing technologies, a large number of mRNAs that undergo alternative splicing have been identified in crops and model plants. A number of genome-wide analyses of AS using next-generation sequencing (NGS)-based RNA sequencing (RNA-seq) have been published for model plants and crops such as *Arabidopsis* [23, 24], soybean (*Glycine max*) [25], maize (*Zea mays*) [26], rice (*Oryza sativa*) [15], wheat (*Triticum aestivum* L.) [27], and cotton (*Gossypium raiimondi*) [16]. Single-molecule real-time long-read isoform sequencing has also been used extensively to predict full-length splice isoforms in sorghum (*Sorghum bicolor*) [28], maize (*Zea mays*) [29], and cotton (*Gossypium davidsonii*) [30]. Several methods and pipelines have been developed to detect alternative splicing (AS). These approaches can be categorized into two main groups: event-based and isoform-based quantification [31]. The event-based method represents AS as ratios of a particular event, such as the inclusion of an exon or intron. Programs, such as rMATS [32] and LeafCutter [33] were designed based on an event-based method. In contrast, the isoform-based quantification method estimates the abundances of full-length transcripts and calculates the isoform ratios (the count of one isoform divided by the total isoform counts for the gene).

When such analyses are extended from a small number of samples to a population, the variable AS can be employed as a molecular phenotype and analyzed in association with genetic structure variation to obtain splicing quantitative trait loci (sQTLs). sQTL analysis can be further integrated with GWAS to identify genetic variation loci that are associated with both AS and agronomic traits. Currently, sQTL analyses have only been conducted in a few plants, such as *Arabidopsis* [34], maize [35, 36], and rice [37]. Using the above workflow in maize identified the *trans*-regulatory factor ZmGRP1, which regulates a *trans*-cluster that affects downstream genes. In rice, *OsNUC1* and *OsRAD23* were identified as candidate genes whose transcripts exhibited significant divergence in splicing under salt stress conditions [37].

In this study, transcriptome data from 1-day post anthesis (1-DPA) ovules of CUCP1 were used to elucidate the frequency of IR events and the stability of the transcriptomes of cotton cultivars. Additionally, the study aimed to analyze whether IR is a component of the genetic basis for the regulation of yield traits in cultivated cotton. Cotton fibers develop from epidermal cells on ovules [1], with fiber cell differentiation spanning from 3 days before flowering to 1 day after flowering, this process determines the number of fiber cells [38, 39] and thus constitutes one of the key developmental periods for the formation of yield traits in cotton. eQTL studies have shown that genetic structural variants are associated with gene expression, which in turn is associated with variation in traits such as cotton fiber length [2]. Alternative splicing and transcription are key steps in gene expression regulation, and both occur at the mRNA transcription stage. However, it is unclear whether the frequency of IR in gene transcripts is correlated with variation in gene expression in the crop population. Here, IR events were identified at the population transcriptome level and genome-wide sQTL analysis was conducted to reveal the possible regulatory modules involved with AS events in this cotton population.

## Results

### A workflow to identify the candidate AS events related to complex traits

**Fig. 1:**
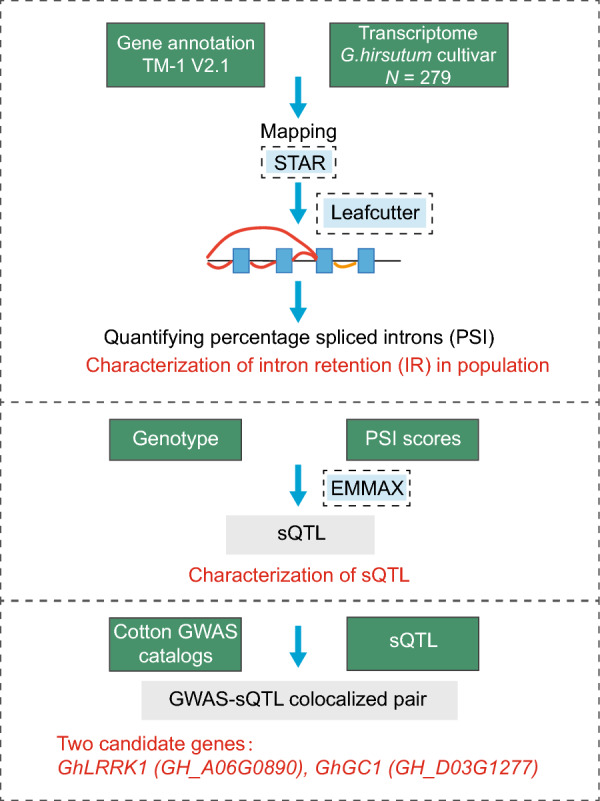
The schematic chart for the pipeline of the study. The data and software used in this study were in solid-line box and dashed box, respectively

Figure 1 illustrates the workflow of the working pipeline to mine the AS events related to complex traits. A total of 279 *Gossypium hirsutum* accessions from CUCP1 (34 wild or local varieties and 245 cultivars) were examined in this study (Additional file 2: Table S1) [40]. Transcriptome data from 1-DPA ovules were aligned to the gene annotations of the upland cotton, genetic standard line Texas Marker-1 (TM-1) [41]. And intron retentions (IRs) were quantified using percent spliced in index (PSI), a common and intuitive ratio for splicing events, using the LeafCutter software [33]. The IR events in population were then characterized and subjected for sQTL analysis using the EMMAX software [42]. To navigate the potential functional AS events related to complex traits, the GWAS catalog were retrieved from our previous study [7] for colocalization analysis.

### Genome-wide identification and characterization of IR in a cotton population

**Fig. 2:**
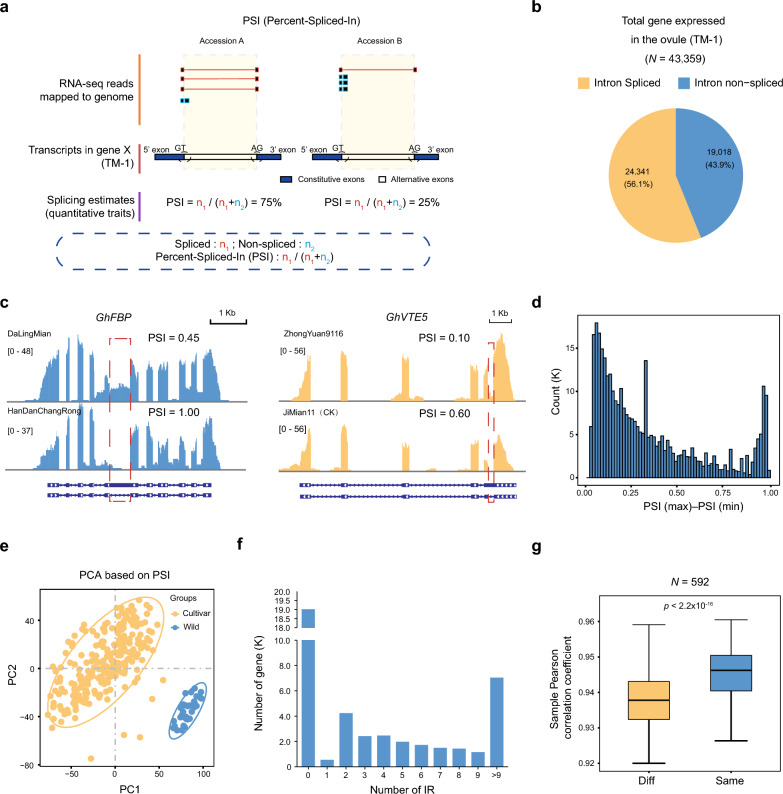
Quantification of intron retention (IR) in an upland cotton population. **a** Percent spliced in (PSI) scores were calculated by taking the ratios of junctions observed over annotated splice junctions. The two examples show one intron region in two different accessions. n_2_ is the count of junction reads for the single intron, and n_1_ is the count for surrounding exons. **b** Pie plot showing the distribution of IR-coupled genes of TM-1 expressed genes. **c** IGV visualization of intron retention in two genes with different PSI scores. The red dotted box indicates the intron of interest; PSI was calculated in two accessions. **d** The distribution of PSI difference (PSI (max) − PSI [4]) among the population. **e** Principal component analysis (PCA) based on PSI scores, which shows a distinct separation of wild and cultivar groups. **f** Number of IR events identified for each gene. The x-axis and y-axis represent the number of IR events identified per gene and the number of genes in each group, respectively. **g** Box plot of Pearson’s correlation coefficient (PCC) for the PSI scores of different samples. “Same” and “diff” indicate whether the two samples are biological replicates. Boxes span the first to third quartiles and center lines indicate the second quartile (median)

PSI was calculated for each intron by dividing the number of transcript elements presented by the total number of reads covering the splicing event, yielding scores ranging from 0 to 1 (A value of 0 indicated that the intron has not been spliced at all) [43] (Fig. 2a). A total of 341,491 IR events and a total of 43,359 genes were expressed in the 1-DPA ovule of TM-1. 24,341 (56.14%) of genes were identified to harbor IR events (Fig. 2b). Figure 2c showed example of IRs and its corresponding PSI scores. The per-site PSI range (maximum–minimum) across the sequenced population largely concentrated in the interval of 0.02–0.99, with a peak at 0.07 (Fig. 2d), indicating that most IR events do not vary within the population.

In addition, principal component analysis (PCA) of PSI profiles revealed a distinct pattern distinguishing wild from cultivar accessions (Fig. 2e), suggesting that the IR phenomenon was under selection during the domestication of upland cotton. Overall, 23,946 (55.22%) of expressed genes were found to undergo two or more IR events (Fig. 2f). The Pearson correlation coefficients (PCCs) of the PSI between the two biological replicates (mean r = 0.95) were significantly higher than those of different accessions. (mean r = 0.93, *p*-value < 2.2 × 10^–16^, Mann–Whitney test, Fig. 2g), a result for which both wild and cultivated subgroups were in high agreement (Additional file 1: Figs. S1 and S2); this indicates that the sites at which intron retention-associated splicing occurs are specific towards each accession in this population.

### Genome-wide association study of IR-based PSI in cotton ovule

To improve the computation efficiency for population-wide genome scans, the identified IRs were filtered according to the following criteria: (i) high expression (FPKM ≥ 1 at the gene level for 95% of germplasms in the population); (ii) high variation in PSI (for each IR event, coefficient of variation of PSI > 0.1 and standard deviation > 0.1 in the population); (iii) intron lengths < 5000 bp. At the end, a total 29,492 IR events were retained for genome-wide association analysis (GWAS) (Additional file 1: Fig. S3).

**Fig. 3:**
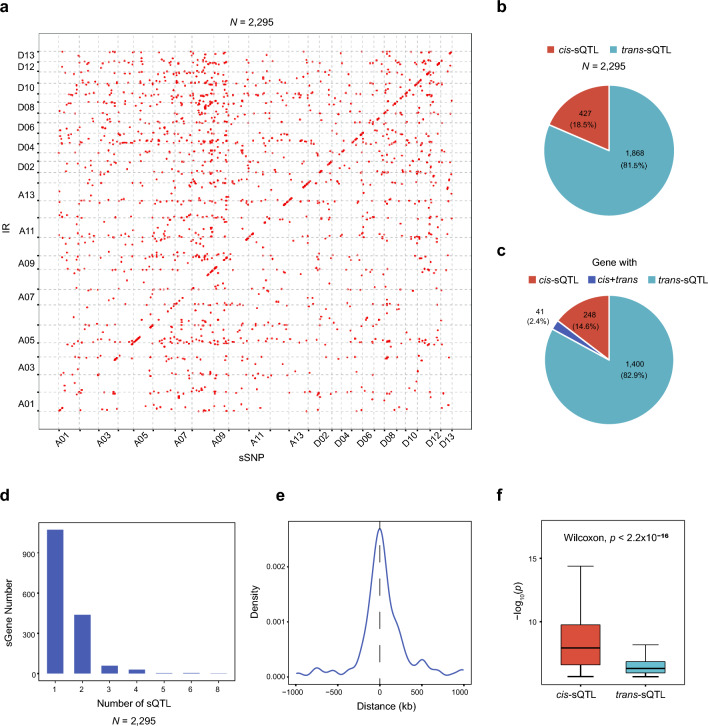
sQTL mapping and assessment. **a** Scater plot showing the number of sQTLs. The *x*-axis and *y*-axis indicate the physical position of the lead SNP of the sQTL and its associated splice junction, respectively. Each dot represents a sQTL. Dots on the diagonal line indicate intrachromosomal associations. **b** Pie chart of *cis*- and *trans*-sQTLs. **c** Pie chart of IR-coupled genes with *cis*- and/or *trans-*sQTLs. **d** Histogramm of the sQTLs identified for each IR-coupled gene. The *x*-axis and *y*-axis represent the number of sQTLs identified for each gene and the number of genes in each group, respectively. **e** The density distribution of *cis*-sQTLs along the span between associated *cis*-SNP and IR splicing sites. **f** Significances of *cis*-sQTLs and *trans*-sQTLs. For boxplots, the lower and upper horizon lines are the minimum and maximum values of − log10 (*p*), respectively; the boxes span from the first to third quartiles; and the center line indicates the second quartile (median)

GWAS of these 29,492 IR sites with 1,186,673 biallelic SNPs (MAF > 0.05, missing rate < 20%) was conducted with Efficient Mixed Model Analysis Expedited (EMMAX), applying a cutoff of *p*-value < 2.18 × 10^–6^ for genome-wide significance. In total, 2295 sQTLs (Additional file 3: Table S2) were obtained, regulating 2199 IR events in 1607 genes (Fig. 3a, Additional file 1: Fig. S4). The sQTL-featured SNPs associated with IR events were termed sSNPs. These sQTLs were categorized according to the distance between sQTL and sSNP: those with a separation of less than 1 Mb were defined as *cis*-sQTLs, and all others as *tran*s-sQTLs. Of the 2295 sQTLs found, 427 were *cis*-sQTLs and 1868 were *trans*-sQTLs (Fig. 3b). The *cis*-sQTLs were collectively associated with 1607 genes (Fig. 3c). As mentioned above, most multi-exon genes subject to IR have more than one IR event (Fig. 2f); additionally, an average of three sQTLs was mapped for each gene (Fig. 3d). For *cis*-sQTLs, the associated sSNPs were predominantly distributed in adjacent genes and enriched for proximity to transcription start or termination sites (TSS or TTS) (Fig. 3e). The significance of each *cis*-sQTLs was greater than that of *trans*-sQTLs. (*p*-value = 7.75 × 10^–7^, Wilcoxon test) (Fig. 3f). *Trans*-sQTL effect power tended to be smaller, thus we focused on *cis*-sQTLs for further analysis.

### The relationship between IR and gene expression in cotton population

**Fig. 4:**
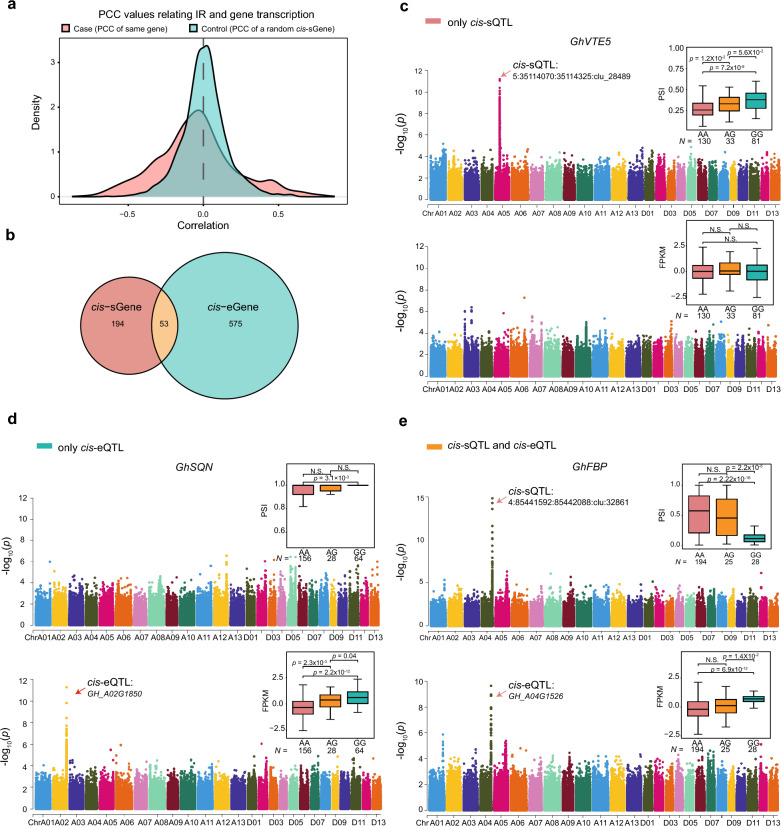
Relative independence in the genetic control of *cis*-sQTLs and *cis*-eQTLs. **a** Distribution of Pearson’s correlation values relating PSI with gene expression. Case: PCC was calculated for PSI and expression of the same gene. Control: PCC was calculated for PSI and expression of a randomly selected gene. **b** Venn diagram showing the overlap between genes with *cis*-sGenes and those with *cis*-eGenes. From **c** to **e**, Manhattan plots show GWAS results for splicing level [51] and overall expression level of each gene (bottom). For boxplots, the lower and upper horizon lines are minimum and maximum values, respectively; the boxes span from the first to third quartiles; and the center line indicates the second quartile (median). *p*-values were calculated by two-sided Student’s *t*-test. **c** Manhattan plot showing a gene (*GhVTE5*/*GH_A05G2930*) detected to have a *cis*-sQTL without effect on expression level. Box plots display the association of sSNP haplotype (A05:35150218; AA, GG, and AG) with splicing level as indicated by PSI score [51] and with total mRNA level (bottom). **d** Manhattan plot showing a gene (*GhSQN* /*GH_A02G1850*) detected to have a *cis*-eQTL without effect on splicing. Box plots display the association of sSNP haplotype (A02:106438154; AA, GG, and AG) with splicing level as indicated by PSI score [51] and with total mRNA level (bottom). **e** Manhattan plot showing a gene (*GhFBP*/*GH_A04G1526*) detected to have both a *cis*-sQTL and *cis*-eQTL. Box plots display the association of sSNP haplotype (A04:85359924; AA, GG, and AG) with splicing level as indicated by PSI score [51] and with total mRNA level (bottom)

eQTL analysis using the same transcriptome data has been completed by Zhao et al., in which a total of 12,207 eQTLs were identified [40]. To investigate whether there is a co-regulatory relationship between population-wide IR and gene transcription, PCC was calculated for PSI values and the expression of the corresponding gene within the population. The correlation between PSI scores and the expression of randomly selected genes was used as a control. This analysis revealed that PSI of a gene was not typically correlated with its expression, with only 3.6% of PSI-gene pairs exhibiting substantial correlation (PCC > 0.6) (Fig. 4a and Additional file 1: Fig. S5).

The genes found here to be associated by a *cis*-sQTL or *cis*-eQTL can be categorized into three types according to the effects of sQTL and eQTL on the same gene, *cis*-sQTL only, *cis*-eQTL only and under both *cis*-sQTL/eQTL. To determine whether sQTLs and eQTLs are *cis*-regulated by the same genetic loci, co-localization of the two QTL types was examined. A *cis*-sQTL and a *cis*-eQTL in the same gene were defined to co-localized, if their corresponding regulatory SNPs were within 100 Kb of each other, and they were in linkage disequilibrium (LD, *r*^2^ > 0.1). There were 194, 575, and 53 genes detected for the above three types, respectively (Fig. 4b). Of the 247 *cis*-sQTL-regulated genes, merely 53 (21.4%) *cis*-sGene were found to co-localize with significant *cis*-eQTLs (Fig. 4b), suggesting that the majority of mRNA alternative splicing are independent to eQTLs. Similar trend was observed in maize [35] and rice [37]. This implies that AS of mRNA may provide a novel avenue for further study of SNP-phenotype associations and investigation of phenotypic genetic mechanisms.

For example, *GhVTE5* (*GH_A05G2930*), a gene associated with vitamin E synthesis, is a significant *cis*-sQTL detected in the fourth intron (A05:35114070:35114325:clu_28489, *p*-value = 1.01 × 10^–8^). The associated SNP (A05:35150218) has two haplotypes, GG and AA. PSI values were significantly higher for the GG haplotype than the AA haplotype (*p*-value = 7.2 × 10^–9^, Student’s *t*-test), however no significant transcriptional variation was detected on this gene (Fig. 4c). *GhSQN* (*GH_A02G1850*), encoding cyclophilin 40, achieved significance (*p*-value = 2.96 × 10^–11^) for genetic association with gene expression but is not regulated by any identified sQTL (Fig. 4d). The gene encoding fructose-1,6-bisphosphatase, *GhFBP* (*GH_A04G1526*), which is associated with a *cis*-sQTL (A04:85441592:85442088:clu_32861, *p*-value = 4.85 × 10^–7^) and a *cis*-eQTL (*p*-value = 5.69 × 10^–11^). The AA haplotype of the linked eSNP was associated with higher expression (*p*-value = 6.9 × 10^–12^, Student’s *t*-test (Fig. 4e), and it was in linkage disequilibrium with the sSNP (*p*-value = 5.69 × 10^–11^).

### Association analysis of IR events as markers for agronomic traits

To investigate the role of sQTLs in determining agronomic traits in cotton, we further co-localized the sQTL with GWAS loci [7]. The 187 independent GWAS loci from Fang et al.’s study were represented as pSNPs, which are phenotypic SNPs associated with agronomic traits [7]. This analysis yielded 32 functional *cis*-sQTL loci, of which 30 were associated with yield traits and 2 with fiber quality traits (Additional file 4: Table S3).

**Fig. 5:**
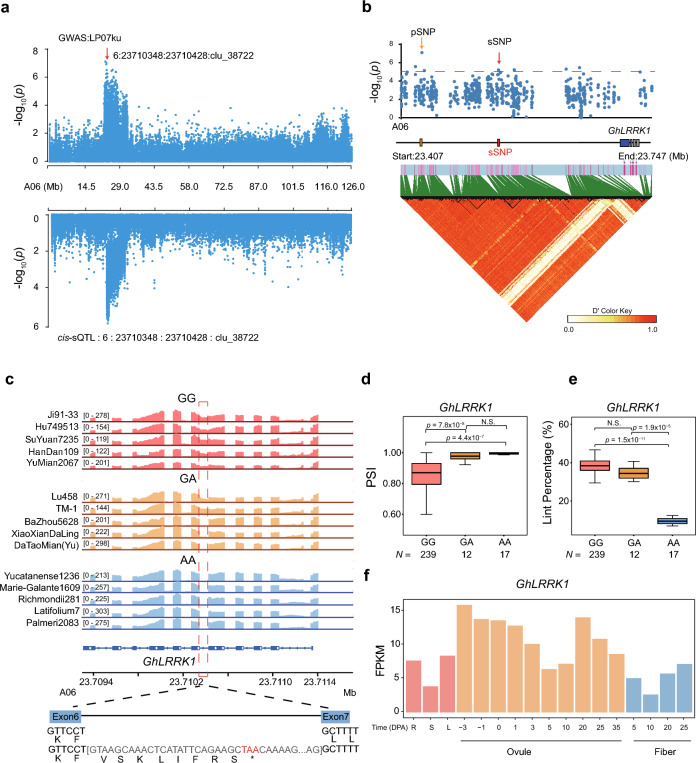
The IR event in *GhLRRK1* (*GH_A06G0890*) and its association with cotton lint percentage (LP). **a** Manhattan plots for lint percentage (LP) based on GWAS [51] and sQTL mapping (bottom). The GWAS plot shows a signal on chromosome A06 that is associated with lint percentage, and the *cis*-sQTL plot a signal in *GhLRRK1* (A06:23710348:23710428:clu_38722). **b** Local Manhattan plot [51] and LD heat map (bottom) for the sSNP (A06:23513733). The arrowhead indicates the SNP in the candidate gene. The horizontal dashed line indicates the significance threshold (*p*-value < 1 × 10^−5^). Red box shows the sSNP locus, the orange box shows the pSNP locus, the blue box shows the *GhLRRK1.* **c** Visualization of *GhLRRK1* transcript structure and genotype-specific splicing (GG, GA, and AA). The IGV screenshot [51] shows the total read numbers for each junction among individuals of different haplotypes. The structural schematic (bottom) shows the impact of IR on the GhLRRK1 protein. Retention of the 6th intron alters the predicted protein sequence and produces a premature stop codon (PTC). Red dotted box shows the IR locus. **d** Boxplot showing the difference of PSI explained by the haplotype (GG, GA, and AA) of sSNP A06:23513733. Boxes in box plots span from the first to third quartiles, and center lines indicate the second quartile (median). *p*-values were calculated by two-sided Student’s *t*-test. **e** Boxplot showing the difference in lint percentage (%) explained by the haplotype (GG, GA, and AA) of sSNP A06:23513733. Boxes in box plots span from the first to third quartiles, and center lines indicate the second quartile (median). *p*-values were calculated by two-sided Student’s t-test. **f** Transcriptomic level of *GhLRRK1* in different tissues, including R (root), S (stem), and L (leaf), during ovule and fiber development, based on the FPKM values from a single experiment

One locus on chromosome A06 featured by a lead pSNP (A06:23741067, Fig. 5a) is significantly associated with LP (*p*-value = 1.24 × 10^–6^). The GWAS signal for this locus colocalizes with a *cis*-sQTL (A06:23710348:23710428:clu_38722,* p*-value = 1.35 × 10^−6^) featured by a sSNP (A06:23513733) (Fig. 5a). The *cis*-sQTL is located in the sixth intron of *GhLRRK1* (*GH_A060890*), a gene encoding a leucine-rich repeat protein. Analysis of pairwise LD and *r*^2^ showed the corresponding pSNP and sSNP to be in high LD; moreover, the entire gene fell in a single LD block (Fig. 5b). Next, the mRNA splice junction was visualized in Integrative Genomics Viewer (IGV). As illustrated in Fig. 5c, this revealed differences in intron retention at this locus among different accessions. The two haplotypes were each associated with distinct rates of IR. This is consistent with PSI determinations, accessions with intron retention (GG haplotype) to have significantly lower PSI scores than those with no retention (AA haplotype) (*p* < 4.4 × 10^–7^, Student’s *t*-test, Fig. 5d). Protein sequence translation prediction was then carried out for both alleles, which determined that retention of the sixth intron (A06:23710348:2310428:clu38722) introduced a PTC and caused a 125-amino-acid deletion in the protein. In addition, the LP of the homozygous genotypes were analyzed, which reveal accessions with intron retention (GG haplotype exhibited significantly increased LP (~ 26% greater, *p*-value < 1.5 × 10^−11^, Student’s *t*-test; Fig. 5e). This suggests that retention of the sixth intron of *GhLRRK1* is positively correlated with cotton yield. Notably, accessions with AA haplotype at this locus are dominant in all wild-type cottons while those with GG haplotype (freq_(GG) = 0.79) are the majority in the cultivated accessions. In addition, this gene is near to a putatively selected region of upland cotton chromosome A06 identified by Yuan [44], so it is feasible that this difference in intron retention may be related to cotton domestication. RNA-seq data from different stages of fiber development showed *GhLRRK1* to be highly expressed in ovules and fibers (Fig. 5f), and RT-PCR analysis of 1-DPA ovules representing the two haplotypes (AA and GG) confirmed the retention of this intron in accessions with GG haplotype (Additional file 1: Fig. S6). Therefore, *GhLRRK1* is presumed to be a novel gene that controls fiber development by its alternative splicing.

**Fig. 6:**
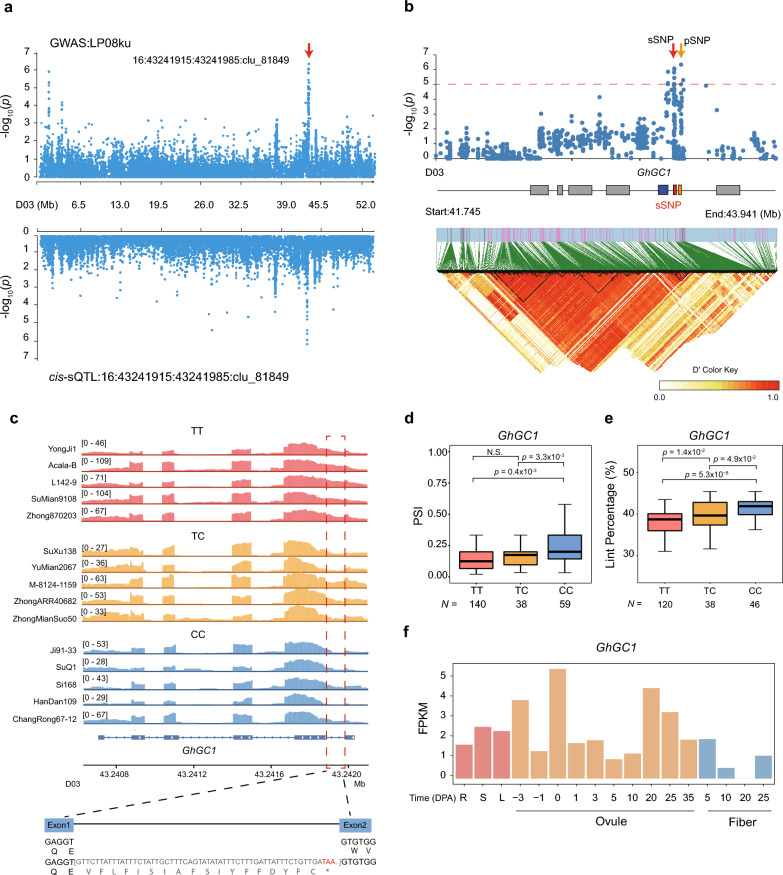
The IR event in *GhGC1 (GH_D03G1277)* and its association with cotton lint percentage (LP). **a** Manhattan plots for LP based on GWAS [51] and sQTL mapping (bottom). Each dot represents a single SNP. The GWAS plot shows a signal on chromosome D03 that is associated with lint percentage, and the *cis*-sQTL plot shows a signal in *GhGC1* (D03:43241915:43241985:clu_81849). **b** Local Manhattan plot [51] and LD heat map (bottom) for the sSNP (D03:43244243). The arrowhead indicates the SNP in the candidate gene. The horizontal dashed line indicates the significance threshold (*p*-value < 1 × 10^−5^). Red box shows the sSNP locus, the orange box shows the pSNP locus, the blue box shows the *GhGC1*. **c** Visualization of *GhGC1* transcript structure and genotype-specific splicing (TT, TC, and CC). The IGV screenshot [51] shows the total read numbers for each junction among individuals of different haplotypes. The structural schematic (bottom) shows the impact of IR on the GhGC1 protein. Retention of the 1st intron alters the predicted protein sequence and produces a premature stop codon (PTC). The red dotted box shows the IR locus. **d** Boxplot showing the difference of PSI explained by the haplotype (TT, TC, and CC) of sSNP D03:43244243. Boxes in box plots span from the first to third quartiles, and center lines indicate the second quartile (median). *p*-values were calculated by two-sided Student’s *t*-test. **e** Boxplot showing the difference in lint percentage (%) explained by the haplotype (TT, TC, and CC) of sSNP D03:43244243. Boxes in box plots span from the first to third quartiles, and center lines indicate the second quartile (median). *p*-values were calculated by two-sided Student’s *t*-test. **f** Transcriptomic level of *GhGC1* in different tissues, including R (root), S (stem), and L (leaf), during ovule and fiber development, based on the FPKM values from a single experiment

As another example, a significant pSNP (D03:43283363) associated with the LP GWAS locus on chromosome D03 (*p*-value = 1.24 × 10^–6^) was co-localized with a *cis*-sQTL locus (D03:43241915:43241985:clu_81849, *p*-value = 7.75 × 10^–7^) (Fig. 6a). Analysis of pairwise LD and *r*^2^ showed that the sSNP (D03:43244243), pSNP (D03:43283363), and gene *GhGC1* (*GH_D031277*) were all in an LD block (Fig. 6b). Visualization of read data with IGV showed that the transcriptional structure at this position varied across the population (Fig. 6c), with the first intron of *GhGC1* being differentially retained: generally included with the TT haplotype and spliced out with the CC haplotype (Fig. 6d); this is consistent with PSI determinations, with the TT genotype corresponding to significantly lower PSI score than the CC haplotype (*p*-value < 0.4 × 10^–3^, Student’s *t*-test). Prediction of the protein sequence of the two transcript isoforms revealed retention of intron one (D03:43241915:43241985:clu_81849) to produce a frameshift mutation and a PTC (Fig. 6c). Notably, accessions with no intron retention (CC haplotype) exhibited 4% greater in LP compared to those with retention (TT haplotype) (*p*-value < 5.3 × 10^–8^, Student’s *t*-test; Fig. 6e), suggesting a positive correlation between splicing of this intron and modest cotton yield enhancement. RNA-seq data from different stages of fiber development showed high expression of *GhGC1* in ovules (Fig. 6f), and RT-PCR confirmed differences in retention of the first intron between the two haplotypes (Additional file 1: Fig. S7). Thus, *GhGC1* is speculated to be a candidate gene regulating fiber lint.

Collectively, these findings suggest that IR is stable and genetically variable in cotton cultivar populations. Integrative analysis of sQTL and GWAS results revealed a significant association of IR variation with cotton fiber traits, and further validate such genes as candidates for causing phenotypic variation. Because these trait-associated sQTLs were obtained from analysis of genetic structure variation in natural populations, they constitute a considerable genetic resource for uncovering candidate genes for breeding applications.

### Identification of sQTLs harbored in transcription factors

**Fig. 7:**
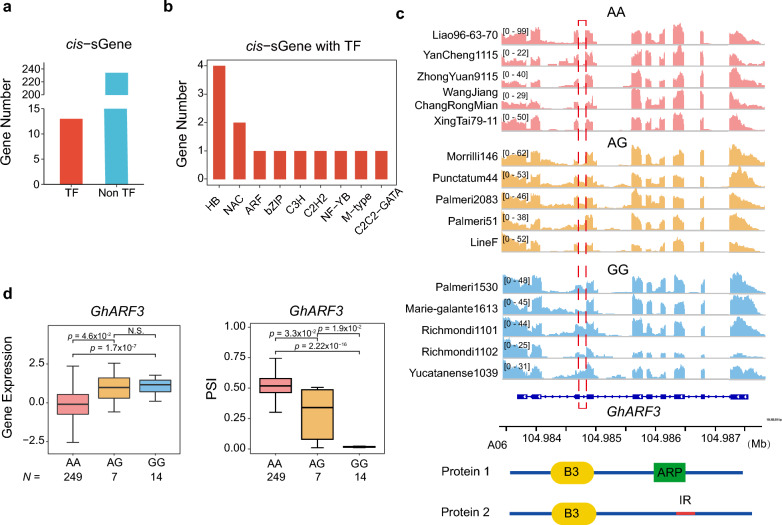
Impacts of IR on transcription factors. **a** Bar plot showing the number of *cis*-sQTL genes encoding transcription factors. **b** Bar plot showing the number of *cis*-sQTL genes belonging to different TF families. **c** An example of a gene (*GhARF3*/*GH_A06G1554)* with a significant *cis*-sQTL (A06:104984673:104984791:clu_39823) encoding an ARF transcription factor. The IGV screenshot [51] shows the total read numbers for each junction among individuals of different haplotypes, red dotted box shows the IR locus. The structural schematic (bottom) shows the impact of IR on the GhARF3 protein. For the transcript model, blue boxes indicate coding sequence, and lines indicate introns. In the bottom, protein 2 denotes the new structure produced by IR, which lacks the ARP domain present in the original protein 1. **d** Distribution of gene expression (left) and PSI according to haplotype (AA, AG, and GG) of sSNP A06:105487159. Data are presented as median (minimum to maximum). *p*-values were calculated by two-sided Student’s *t*-test

Several studies have shown that the occurrence of IR in genes encoding transcription factors may result in the loss of protein activity due to the deletion of key functional domains [45, 46], and that IR can act as an important mechanism for regulating DNA binding and transcriptional activity [47]. To explore the possibility of IR effects on transcription factor function, a predictive analysis was performed on the 5409 transcription factor genes annotated in TM-1 [41]. A total of 13 genes encoding transcription factors from nine families (HB, NAC, ARF, bZIP, C3H, C2H2, NF-YB, M-type, and C2C2-GATA) were found to be represented by the *cis*-sQTL genes (Fig. 7a and b, Additional file 5: Table S4).

As one example, there is a significant *cis*-sQTL (A06:10484673:10484791:clu_39823) in the auxin response factor (ARF) gene *GhARF3* (*GH_A06G1554*), which encodes three conserved structural domains: a plant-specific B3 DNA-binding domain at the N-terminal end, an Auxin_resp (ARP) domain and an unnamed conserved domain at the C-terminal end. Visualization in IGV confirmed a difference in retention of the gene’s third intron between the two sSNP alleles (Fig. 7c). In plants with the AA haplotype, this intron region is almost uniformly retained, whereas in those with the GG haplotype, it is spliced. This observed difference was consistent with the calculated PSI scores, and achieved significance (*p*-value < 2.22 × 10^–16^, Student’s *t*-test). Retention of the third intron (GG haplotype) results in loss of the ARP domain (Fig. 7c), and overall gene expression is significantly increased (GG, mean = 1.25; AA, mean = − 0.16) (*p*-value < 1.7 × 10^–7^, Student’s *t*-test; Fig. 7d). RNA-seq showed *GhARF3* to be highly expressed in ovules (Additional file 1: Fig. S8). Two accessions, one representing each haplotype, were selected for RT-PCR validation and confirmed presence of the splicing variant in the third intron of *GhARF3* (Additional file 1: Fig. S9). In summary, a naturally occurring alternative splicing variant (IR) of a transcription factor could impact protein function via producing an early termination codons or disrupting structural domains.

### IR-induced regulation potentials by miRNA targeting

**Fig. 8:**
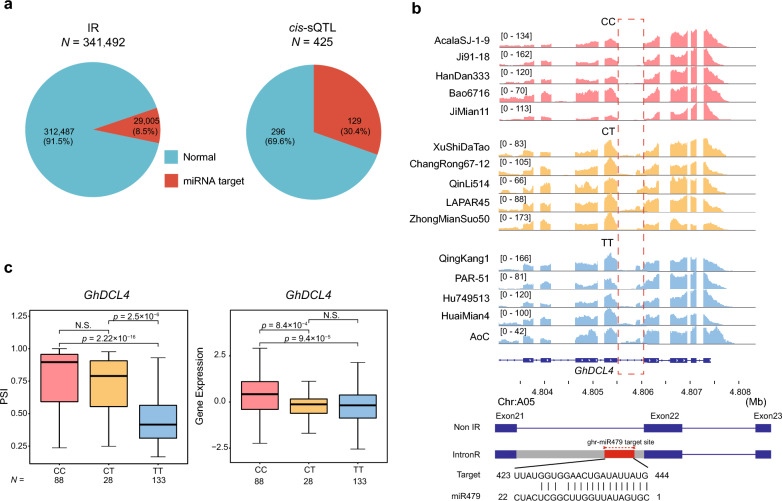
Association of IR with miRNA-mediated regulation. **a** Pie plot showing the distribution of potential miRNA target sites in IR-coupled genes (left) and *cis*-sQTL genes [2]. **b** An example of the IR-coupled miRNA mechanism. *GhDCL4* (*GH_A05G0514*) was detected to have a significant *cis*-sQTL (A05:4805506:4806040:clu_24377). The IGV screenshot [51] shows the total read numbers for each junction among individuals of different haplotypes, red dotted box shows the IR locus. The transcript model (bottom) shows the impact of IR on predicted miRNA targeting of *GhDCL4*. Blue boxes indicate the coding sequence, and lines indicate introns. Transcripts labeled “IR” are produced from intron retention. The red box indicates the predicted miRNA target site. **c** Distribution of PSI (left) and gene expression [2] according to haplotype (CC, CT, and TT) of sSNP A05:4938322. Boxes in box plots span from the first to third quartiles, and center lines indicate the second quartile (median). *p-*values were calculated by two-sided Student’s *t*-test

Increasing evidence supports that miRNAs play a very important role in the regulation of gene expression [48]. To assess whether the identified intron retention sites might be targets for miRNA, miRNA target prediction was performed for all retained intron sequences using the psRNATarget website and 80 published miRNAs in the upland cotton database [49]. The results showed that 29,005 of the 341,492 IRs (8.5%) have potential miRNA target sites, as did 30.4% (n = 108) of the identified *cis*-sQTLs (Fig. 8a, Additional file 7: Table S6).

To validate the potential miRNA target sites in IRs, the degradome data from the fiber of upland cotton cultivar R15 were adapted [50]. The 29,005 IR sequences with potential miRNA target site were mapped to degradome library and revealed 11,759 cleavage sites (Additional file 1: Fig. S10). Among of which, 131 *cis*-sQTL were validated with cleaved fragments in the degradome library (Additional file 1: Fig. S10). This result confirmed the IR and *cis*-sQTL have an effective potential to be regulated by the predicted miRNA targeting.

As an example, a significant *cis*-sQTL (A05:4805506:4806040:clu_24377, *p*-value = 8.31 × 10^–15^) was detected for the Dicer-like protein *GhDCL4* (*GH_A05G0514*) gene, which is regulated by an sSNP (A13:105444599). Retention of the 21st intron of *GhDCL4* was found to provide a target sequence for miRNA479. Visualization of the site in IGV is consistent with PSI calculations (Fig. 8b and c), as TT show retention of this intron and CC haplotype largely splice it out. The PSI scores of TT haplotype were significantly lower than those of accessions with CC haplotype (*p*-value < 2.22 × 10^–16^, Student’s *t*-test), and the TT haplotype was found to significantly associated with the lower transcriptional activity of the gene (*p*-value < 9.4 × 10^–5^, Student’s *t*-test, Fig. 8c). RNA-seq showed *GhDCL4* to be highly expressed in ovules (Additional file 1: Fig. S11) and RT-PCR confirmed that the 21st intron was alternatively spliced in accordance with haplotypes (Additional file 1: Fig. S12). Importantly, Dicer-like proteins are a class of exonuclease enzymes whose function is to cleave double-stranded RNA into small RNAs of 21 or 24 nt, including miRNAs. These proteins play important roles in RNA silencing mechanisms, and serve to control gene transcription regulation and antiviral protection in plants. Taken together, these findings support that intron retention might be prevalently coupled with miRNA regulation.

## Discussion

Improving fiber yield is one of the main objectives of cotton breeding, and the flow of GWAS can identify candidate trait loci associated with cotton yield [7]. However, moving from association to the identification of specific causal genes and their biological mechanisms remains a major challenge. In plants, IR has been shown to be the predominant and conserved AS type, playing an important role in plant growth, development, and stress response. Current software tools applied to NGS data to identify AS events include MISO [51], rMATS [32], and Leafcutter [33]; such analysis is followed by experimental verification such as with RT-PCR. However, despite the potential for providing insight into the mechanisms of genetic regulation of phenotypic traits, few studies have applied population transcriptome analysis to dissect the relationship between AS of mRNAs and agronomic traits in crops. Although splicing variation and complex transcript variations have been identified in individual plants, transcript isoform variation remains poorly understood in natural populations. In this study, we identified IR events in 1-DPA ovules using transcriptome data from a population of 279 upland cotton accessions, and conducted a genome-wide analysis of the distribution and potential function of IR. A total of 341,491 IR events were identified in this study using a population transcriptome. As this study employed a population transcriptome, the number of IR events identified was much larger than those studies mining AS events in a single individual plant. The subsequent genome-wide association analysis of IR event frequency with genetic structural variation in the population identified 2295 sQTLs.

Recent studies have found that genetic variants play roles in regulating gene expression in a population, and a proportion of eQTLs are associated with agronomic traits [8]. Here, we further analyzed the correlations between eQTLs and sQTLs. Our findings suggest that the probability of simultaneous effects of genetic structure variation on transcriptional activity and alternative splicing of a given gene is low, which in turn implies that elucidation of *cis*-sQTLs could be an effective means of uncovering genetic variation that influences agronomic traits.

LP in cotton is a complex quantitative trait associated with seed size and lint yield, and is known to be regulated by a variety of genes [4]. In this study, our integrated sQTL and GWAS revealed two causal genes that influence LP, *GhLRRK1* and *GhGC1*, both of which are regulated by *cis*-sQTLs and show significant differences in LP between their respective splicing variants. LRR-RLK family members are important in regulating plant growth and development and stress response, and prior studies of the family member *SERK* suggest it to have a regulatory role in ovule development in both *Arabidopsis* [52] and maize [53]. Here, we found that intron retention events in the cotton population to alter the structure of *LRRK1*, with the IR positively associated with LP. In addition, the wild cotton does not undergo intron retention, while the cultivated accessions exhibited intron retention and higher LP, suggesting that cotton domestication may have selected for this intron retention locus. Meanwhile, another candidate gene, *GhGC1* is predicted to encode the Golgi structural component golgin, for which no specific function has yet been reported in plants. This gene is also associated with LP, with accessions that retain the first intron (TT haplotype) having low LP, whereas in the natural population, 58.82% (n = 120) of the varieties were low-LP materials with intron retention (TT haplotype). This finding suggests that new high-LP cotton varieties could be cultivated by genetic engineering on this locus.

Another significant *cis*-sQTL (A06:10484673:10484791:clu_39823) was identified in *GhARF3*. ARF family transcription factors regulate the growth hormone response [54], and ARF2b is reported to promote cotton fiber initiation [55]. Han et al. [56] found that *GhARF3* (*Gh_A10G0304*) is associated with cotton fiber length and strength, and may be a key gene for cotton fiber development. The *GhARF3* locus (*GH_D06G1524*) identified in this study is orthologous to *Gh_A10G0304*, according to phylogenetic tree analysis (Additional file 1: Fig. S13), and may also be involved in cotton fiber development.

Collectively, the results from this study support that intron retention have important impact on cotton fiber yield traits. Moreover, it is possible to identify high-yielding cotton through analysis of intron retention and the genetic variation that regulates it, which will be of importance in breeding new high-yielding varieties.

However, there are several limitations to this study. First, the study only used bulk RNA data from 1-DPA ovules of this natural population; future single-cell sequencing and development of spatiotemporal transcriptomes could yield RNA transcript datasets with higher spatial and temporal resolutions that more accurately capture key fiber development genes. Second, although the effects of other confounding factors on AS events were fully considered and tightly controlled for, the sample size in this sQTL analysis was limited (*n* = 279) and only one type of AS was considered, which might not be sufficient for the confident identification of all sQTLs in the population. In future studies, larger sample cohorts with multiple developmental stages represented will be favorable for mining genetic variations that impact gene transcription regulation. Third, although linkages between sQTL-regulated genes and cotton yield traits were identified in this study, differential intron retention was only confirmed by RT-PCR; the effect of differential intron retention on gene function and cotton yield traits as well as the biological process involved all remain to be confirmed in more detail.

In conclusion, as the first population-level sQTL analysis in upland cotton, this study provided a fundamental resource for exploring AS based on intron retention, resolved the potential functions of sQTLs, proposed for the first time a potential mechanism by which sQTLs can explain phenotypic traits in cotton, and identified two candidate genes associated with LP for subsequent studies on cotton trait formation. Subsequent studies investigating the mechanisms of cotton trait formation will aid our understanding of the role of alternative splicing and genetic variation in this process and identify candidate loci for use in cotton breeding.

## Conclusion

A pipeline for identify the IR events, the predominant type of AS in plants, were was established at the population transcriptome level. And genome-wide sQTL analysis was conducted according to the PSI on IRs. This study provides population-level genetic clues that AS of mRNA has impacts on crop yield, along with functional sQTLs as new genetic resources for cotton precision breeding.

## Materials and methods

### Plant materials and sampling

In total, 279 accessions of CUCP1, upland cotton population were collected from the Institute of Cotton Research at CAAS, including 34 wild/landrace *Gossypium hirsutum* accessions and 245 core germplasm samples (Additional file 2: Table S1). The 245 core germplasm accessions and 32 of the wild accessions had whole-genome sequencing were previously genotyped by our lab [7]. RNA-seq was previously performed on ovule tissue at 1 day post anthesis (DPA) for all 279 accessions [40], which transcriptome data was used for IR analysis in this study. Detailed information on the transcriptome sequencing can be found in the previous study. In brief, 16–18 plants were grown for each accession, and the collected 1-DPA ovules were bulked for total RNA extraction and sequencing in two replications.

The 279 accessions were grown and phenotyped (seed index [SI], boll weight [BW], boll number [BN], lint percentage [LP], fiber elongation [FE], fiber micronaire [FM], fiber length [FL], and fiber strength [FS]) for 3 years (2007, 2008, and 2009) in three environments: the city of Anyang (AY) in the Yellow River cotton-growing area, the city of Nanjing [57] in the Yangtze River cotton-growing area, and Kuero in the Northwestern cotton-growing area [7].

### SNP identification and annotation

Quality control and filtering of the short sequencing reads was carried out using fastp (V 0.12.2) with default parameters [58]. The remaining clean data were mapped to the allotetraploid cotton TM-1 (V 2.1) genome [41] with the STAR software [59]. The mapping results were converted to BAM files and sorted using SAMtools (V 1.16) [60]. Duplicated reads were filtered using Picard (http://picard.sourceforge.net), and only reads with a unique mapping were used for SNP calling using the Genome Analysis Toolkit (GATK) (v3.7) [61]. Only those SNPs that were supported by GATK were retained. For GWAS and sQTL analyses, SNPs with a minor allele frequency of less than 5% were filtered using VCFtools (V 0.1.13) [62]. Missing genotype data were imputed using Beagle [63]. The ANNOVAR software was used to annotate the remaining SNPs [64]. Ultimately, 1,186,673 autosomal SNPs were identified.

### Identification of IR

RNA-seq reads were mapped using STAR (V 2.5.2) to TM-1 (V 2.1) genome annotations [41]. To quantify expression of intron-retaining transcripts, we used LeafCutter [33], which does not use annotation for splice junction quantification, potentially allowing for the discovery of uncharacterized junctions along with annotated splice sites. Splice junction counts were required to have five reads in at least 25% of samples; this filtering yielded a set of 341,491 IR events.

### sQTL mapping

An integrative sQTL analysis was conducted for variant genotypes and IR events by using EMMAX with a mixed linear model and default parameter [42]. In total, 29,492 high quality IR events were selected for further population, the flowchart for which is illustrated in Additional file 1: Fig. S3: high expression of gene (FPKM ≥ 1); high variation in PSI (for each IR event, coefficient of variation of PSI > 0.1 and standard deviation > 0.1 in the population); and intron length < 5000 bp. The PSI of each IR event was normalized using QQ-normal in R [65]. To control potential confounding factors, population structure and a kinship matrix were incorporated. Population structure (PCs) was calculated using GCTA (V 1.92.1) [66], and the first two PCs were included as covariates in the association analysis. Kinship matrices were obtained using the emmax-kin function of EMMAX with parameters (-v -d 10) [42]. Pairwise linkage disequilibrium (LD) and *r*^2^ values were evaluated by plink (V 1.90) with parameters (-r2 -l -window 99999) [67]. A unique sQTL was defined when the associated SNP was not in LD (*r*^2^ < 0.1) with any other SNPs on the same chromosome that were also associated with the target gene. Finally, the threshold of genome-wide significance was taken as the Bonferroni-corrected *p*-value < 2.18 × 10^–6^ suggested by GEC [68], under which a total of 2295 sQTLs were considered statistically significant.

### Gene Ontology analysis

Gene Ontology (GO) term enrichment analysis of genes associated with *cis*-sQTLs was performed using the R package ClusterProfiler [69]. All genes in the cotton genome were used as background. The GO annotation for cotton was obtained from TM-1 v2.1 [41]. Terms were considered significantly enriched at a corrected (after false discovery rate adjustment) *p*-value < 0.05.

### Functional effect of *cis*-sQTLs in transcription factors

The DNA sequences of candidate genes were extracted from the reference genome TM-1 v2.1, and the corresponding protein sequence was predicted through the NCBI website (https://www.ncbi.nlm.nih.gov/Structure/cdd/wrpsb.cgi) to identify conserved structural domains [70]. The position of the IR loci was used to determine whether the site had an effect on any structural domain of the transcription factor.

### miRNA target prediction

To determine whether IR is conducive to miRNA-mediated regulation, the sequences of identified retained introns were extracted from the reference genome (TM-1. v2). Firstly, sequences were queried via BLASTn against the *G. hirsutum* miRNAs in miRbase [71] to identify conserved miRNAs; no mismatches were allowed. Targets of miRNAs transcribed from retained intron were then predicted by the web tool psRNA-Target (http://plantgrn.noble.org/psRNATarget/) using default parameters [49]. To reduce false positives when predicting targets, only 80 miRNAs that were annotated as high-confidence mature miRNAs were used for prediction. Sequences having no mismatches of longer than 3 nt with the query sequence.

### RT-qPCR analysis

We used reverse transcription PCR (RT-PCR) to validate the presence of selected previously uncharacterized IR events among CUCP1. RNA was isolated from 1-DPA ovule tissue samples of six individuals with two of each genotype for each SNP. First-strand cDNA was reverse transcribed according to the manufacturer’s instructions (Vazyme) and amplified with primers located in these candidate genes. The primers used are listed in Additional file 6: Table S5. PCR products were electrophoresed on a 2% agarose gel.

## Supplementary Information

**Additional file 1. **Additional figures: **Figs. S1–S13**. **Fig. S1.** Box plot of Pearson’s correlation coefficient (PCC) of PSI scores among wild cotton accessions. **Fig. S2.** Box plot of Pearson’s correlation coefficient (PCC) of PSI scores among cultivar cotton accessions. **Fig. S3.** Filtering of IR events for sQTL mapping. **Fig. S4.** Pie plot showing the IR events regulated by genetic variation. **Fig. S5.** Pie charts showing the distribution of PCC values relating IR and gene transcription. **Fig. S6.** Validation of IR in *GhLRRK1*. **Fig. S7.** Validation of IR in *GhGC1*. **Fig. S8.** Expression of *GhARF3* in different tissues and at different developmental periods, based on FPKM values. **Fig. S9.** Validation of IR in *GhARF3*. **Fig. S10.** Degradome sequencing analysis of the IR. **Fig. S11.** Expression of *GhDCL4* in different tissues and at different developmental periods, based on FPKM values. **Fig. S12.** Validation of IR in *GhDCL4*. **Fig. S13.** Phylogenetic tree representing the relationships among 22 ARF genes of *Gossypium hirsutum* L. and *Arabidopsis thaliana* (L.).

**Additional file 2: Table S1.** Accessions used in this study.

**Additional file 3: Table S2. **sQTL mapping summary.

**Additional file 4: Table S3.** Summary for *cis*-sQTL colocalizing with trait associations.

**Additional file 5: Table S4.** Indentified *cis*-sQTL are affective to transcription factors.

**Additional file 6: Table S5.** Summary of primer sequences used in the study.

**Additional file 7: Table S6.** Summary for cis-sQTL coupled miRNA.

## Data Availability

All RNA sequencing reads have been deposited in the NCBI Short Read Archive (https://www.ncbi.nlm.nih.gov/sra) under Bioproject PRJNA730082. All DNA sequencing reads were retrived from the NCBI Short Read Archive (https://www.ncbi.nlm.nih.gov/sra) under Bioproject PRJNA375965. Sample IDs and metadata can be found in Additional file 2: Table S1.

## References

[1] Basra AS, Malik CP, Bourne GH, Danielli JF (1984). Development of the cotton fiber. International review of cytology.

[2] Chen ZJ, Scheffler BE, Dennis E, Triplett BA, Zhang T, Guo W, Chen X, Stelly DM, Rabinowicz PD, Town CD (2007). Toward sequencing cotton (Gossypium) genomes. Plant Physiol.

[3] Ma Z, He S, Wang X, Sun J, Zhang Y, Zhang G, Wu L, Li Z, Liu Z, Sun G (2018). Resequencing a core collection of upland cotton identifies genomic variation and loci influencing fiber quality and yield. Nat Genet.

[4] Wang M, Tu L, Lin M, Lin Z, Wang P, Yang Q, Ye Z, Shen C, Li J, Zhang L (2017). Asymmetric subgenome selection and cis-regulatory divergence during cotton domestication. Nat Genet.

[5] Zhang Z, Li J, Jamshed M, Shi Y, Liu A, Gong J, Wang S, Zhang J, Sun F, Jia F (2020). Genome-wide quantitative trait loci reveal the genetic basis of cotton fibre quality and yield-related traits in a *Gossypium hirsutum* recombinant inbred line population. Plant Biotechnol J.

[6] Gu Q, Ke H, Liu Z, Lv X, Sun Z, Zhang M, Chen L, Yang J, Zhang Y, Wu L (2020). A high-density genetic map and multiple environmental tests reveal novel quantitative trait loci and candidate genes for fibre quality and yield in cotton. Theor Appl Genet.

[7] Fang L, Wang Q, Hu Y, Jia Y, Chen J, Liu B, Zhang Z, Guan X, Chen S, Zhou B (2017). Genomic analyses in cotton identify signatures of selection and loci associated with fiber quality and yield traits. Nat Genet.

[8] Zhu Z, Zhang F, Hu H, Bakshi A, Robinson MR, Powell JE, Montgomery GW, Goddard ME, Wray NR, Visscher PM, Yang J (2016). Integration of summary data from GWAS and eQTL studies predicts complex trait gene targets. Nat Genet.

[9] Li Z, Wang P, You C, Yu J, Zhang X, Yan F, Ye Z, Shen C, Li B, Guo K (2020). Combined GWAS and eQTL analysis uncovers a genetic regulatory network orchestrating the initiation of secondary cell wall development in cotton. New Phytol.

[10] Walker RL, Ramaswami G, Hartl C, Mancuso N, Gandal MJ, de la Torre-Ubieta L, Pasaniuc B, Stein JL, Geschwind DH (2019). Genetic control of expression and splicing in developing human brain informs disease mechanisms. Cell.

[11] Nilsen TW, Graveley BR (2010). Expansion of the eukaryotic proteome by alternative splicing. Nature.

[12] Kornblihtt AR, Schor IE, Allo M, Dujardin G, Petrillo E, Munoz MJ (2013). Alternative splicing: a pivotal step between eukaryotic transcription and translation. Nat Rev Mol Cell Biol.

[13] Reddy AS, Marquez Y, Kalyna M, Barta A (2013). Complexity of the alternative splicing landscape in plants. Plant Cell.

[14] Wang B-B, Brendel V (2006). Genomewide comparative analysis of alternative splicing in plants. Proc Natl Acad Sci.

[15] Zhang G, Guo G, Hu X, Zhang Y, Li Q, Li R, Zhuang R, Lu Z, He Z, Fang X (2010). Deep RNA sequencing at single base-pair resolution reveals high complexity of the rice transcriptome. Genome Res.

[16] Li Q, Xiao G, Zhu YX (2014). Single-nucleotide resolution mapping of the *Gossypium raimondii* transcriptome reveals a new mechanism for alternative splicing of introns. Mol Plant.

[17] Ottens F, Gehring NH (2016). Physiological and pathophysiological role of nonsense-mediated mRNA decay. Pflugers Arch.

[18] Kalyna M, Simpson CG, Syed NH, Lewandowska D, Marquez Y, Kusenda B, Marshall J, Fuller J, Cardle L, McNicol J (2012). Alternative splicing and nonsense-mediated decay modulate expression of important regulatory genes in Arabidopsis. Nucleic Acids Res.

[19] Remy E, Cabrito TR, Batista RA, Hussein MA, Teixeira MC, Athanasiadis A, Sa-Correia I, Duque P (2014). Intron retention in the 5'UTR of the novel ZIF2 transporter enhances translation to promote zinc tolerance in arabidopsis. PLoS Genet.

[20] Airoldi CA, McKay M, Davies B (2015). MAF2 is regulated by temperature-dependent splicing and represses flowering at low temperatures in parallel with FLM. PLoS ONE.

[21] Sureshkumar S, Dent C, Seleznev A, Tasset C, Balasubramanian S (2016). Nonsense-mediated mRNA decay modulates FLM-dependent thermosensory flowering response in Arabidopsis. Nat Plants.

[22] Liu Y, Liu X, Lin C, Jia X, Zhu H, Song J, Zhang Y (2021). Noncoding RNAs regulate alternative splicing in cancer. J Exp Clin Cancer Res.

[23] Filichkin SA, Priest HD, Givan SA, Shen R, Bryant DW, Fox SE, Wong WK, Mockler TC (2010). Genome-wide mapping of alternative splicing in *Arabidopsis thaliana*. Genome Res.

[24] Li W, Lin WD, Ray P, Lan P, Schmidt W (2013). Genome-wide detection of condition-sensitive alternative splicing in Arabidopsis roots. Plant Physiol.

[25] Shen Y, Zhou Z, Wang Z, Li W, Fang C, Wu M, Ma Y, Liu T, Kong LA, Peng DL, Tian Z (2014). Global dissection of alternative splicing in paleopolyploid soybean. Plant Cell.

[26] Thatcher SR, Zhou W, Leonard A, Wang BB, Beatty M, Zastrow-Hayes G, Zhao X, Baumgarten A, Li B (2014). Genome-wide analysis of alternative splicing in *Zea mays*: landscape and genetic regulation. Plant Cell.

[27] Liu Z, Qin J, Tian X, Xu S, Wang Y, Li H, Wang X, Peng H, Yao Y, Hu Z (2018). Global profiling of alternative splicing landscape responsive to drought, heat and their combination in wheat (*Triticum aestivum* L.). Plant Biotechnol J.

[28] Abdel-Ghany SE, Hamilton M, Jacobi JL, Ngam P, Devitt N, Schilkey F, Ben-Hur A, Reddy AS (2016). A survey of the sorghum transcriptome using single-molecule long reads. Nat Commun.

[29] Wang B, Tseng E, Regulski M, Clark TA, Hon T, Jiao Y, Lu Z, Olson A, Stein JC, Ware D (2016). Unveiling the complexity of the maize transcriptome by single-molecule long-read sequencing. Nat Commun.

[30] Zhu G, Li W, Zhang F, Guo W (2018). RNA-seq analysis reveals alternative splicing under salt stress in cotton, *Gossypium davidsonii*. BMC Genomics.

[31] Castaldi PJ, Abood A, Farber CR, Sheynkman GM (2022). Bridging the splicing gap in human genetics with long-read RNA sequencing: finding the protein isoform drivers of disease. Hum Mol Genet.

[32] Shen S, Park JW, Lu ZX, Lin L, Henry MD, Wu YN, Zhou Q, Xing Y (2014). rMATS: robust and flexible detection of differential alternative splicing from replicate RNA-Seq data. Proc Natl Acad Sci USA.

[33] Li YI, Knowles DA, Humphrey J, Barbeira AN, Dickinson SP, Im HK, Pritchard JK (2018). Annotation-free quantification of RNA splicing using LeafCutter. Nat Genet.

[34] Khokhar W, Hassan MA, Reddy ASN, Chaudhary S, Jabre I, Byrne LJ, Syed NH (2019). Genome-wide identification of splicing quantitative trait loci (sQTLs) in diverse ecotypes of *Arabidopsis thaliana*. Front Plant Sci.

[35] Chen Q, Han Y, Liu H, Wang X, Sun J, Zhao B, Li W, Tian J, Liang Y, Yan J (2018). Genome-wide association analyses reveal the importance of alternative splicing in diversifying gene function and regulating phenotypic variation in maize. Plant Cell.

[36] Mei W, Liu S, Schnable JC, Yeh CT, Springer NM, Schnable PS, Barbazuk WB (2017). A comprehensive analysis of alternative splicing in paleopolyploid maize. Front Plant Sci.

[37] Yu H, Du Q, Campbell M, Yu B, Walia H, Zhang C (2021). Genome-wide discovery of natural variation in pre-mRNA splicing and prioritising causal alternative splicing to salt stress response in rice. New Phytol.

[38] Qin YM, Zhu YX (2011). How cotton fibers elongate: a tale of linear cell-growth mode. Curr Opin Plant Biol.

[39] Prakash P, Srivastava R, Prasad P, Tiwari VK, Kumar A, Pandey S, Sawant SV. Trajectories of cotton fiber initiation: a regulatory perspective. 2020.

[40] Zhao T, Wu H, Wang X, Zhao Y, Wang L, Pan J, Wang S, Han J, Mei H, Lu K, et al. Integration of eQTL and machine learning methods to dissect causal genes with pleiotropic effects in genetic regulation networks of seed cotton yield. BioRxiv. 2023. 10.1016/j.celrep.2023.113111 37676770

[41] Hu Y, Chen J, Fang L, Zhang Z, Ma W, Niu Y, Ju L, Deng J, Zhao T, Lian J (2019). Gossypium barbadense and *Gossypium hirsutum* genomes provide insights into the origin and evolution of allotetraploid cotton. Nat Genet.

[42] Kang HM, Sul JH, Service SK, Zaitlen NA, Kong SY, Freimer NB, Sabatti C, Eskin E (2010). Variance component model to account for sample structure in genome-wide association studies. Nat Genet.

[43] Grabski DF, Broseus L, Kumari B, Rekosh D, Hammarskjold ML, Ritchie W (2021). Intron retention and its impact on gene expression and protein diversity: a review and a practical guide. Wiley Interdiscip Rev RNA.

[44] Yuan D, Grover CE, Hu G, Pan M, Miller ER, Conover JL, Hunt SP, Udall JA, Wendel JF (2021). Parallel and intertwining threads of domestication in allopolyploid cotton. Adv Sci (Weinh).

[45] Seo PJ, Park MJ, Park CM (2013). Alternative splicing of transcription factors in plant responses to low temperature stress: mechanisms and functions. Planta.

[46] Seo PJ, Kim MJ, Ryu JY, Jeong EY, Park CM (2011). Two splice variants of the IDD14 transcription factor competitively form nonfunctional heterodimers which may regulate starch metabolism. Nat Commun.

[47] Wang HL, Zhang Y, Wang T, Yang Q, Yang Y, Li Z, Li B, Wen X, Li W, Yin W (2021). An alternative splicing variant of PtRD26 delays leaf senescence by regulating multiple NAC transcription factors in *Populus*. Plant Cell.

[48] Iwakawa HO, Tomari Y. The functions of microRNAs: mRNA decay and translational repression. Trends Cell Biol. 2015;25:651–665. 10.1016/j.tcb.2015.07.011. 26437588

[49] Dai X, Zhuang Z, Zhao PX (2018). Psrnatarget: a plant small RNA target analysis server (2017 release). Nucleic Acids Res.

[50] Cao JF, Zhao B, Huang CC, Chen ZW, Zhao T, Liu HR, Hu GJ, Shangguan XX, Shan CM, Wang LJ (2020). The miR319-targeted GhTCP4 promotes the transition from cell elongation to wall thickening in cotton fiber. Mol Plant.

[51] Katz Y, Wang ET, Airoldi EM, Burge CB (2010). Analysis and design of RNA sequencing experiments for identifying isoform regulation. Nat Methods.

[52] Hecht VR, Vielle-Calzada J-P, Hartog MV, Schmidt EDL, Boutilier K, Grossniklaus U, de Vries SC (2001). The Arabidopsis *Somatic Embryogenesis Receptor Kinase 1* gene is expressed in developing ovules and embryos and enhances embryogenic competence in culture. Plant Physiol.

[53] Baudino S, Hansen S, Brettschneider R, Hecht VFG, Dresselhaus T, Lörz H, Dumas C, Rogowsky PM (2001). Molecular characterisation of two novel maize LRR receptor-like kinases, which belong to the SERK gene family. Planta.

[54] Guilfoyle TJ, Hagen G (2007). Auxin response factors. Curr Opin Plant Biol.

[55] Zhang X, Cao J, Huang C, Zheng Z, Liu X, Shangguan X, Wang L, Zhang Y, Chen Z (2021). Characterization of cotton ARF factors and the role of GhARF2b in fiber development. BMC Genomics.

[56] Han Z, Hu Y, Tian Q, Cao Y, Si A, Si Z, Zang Y, Xu C, Shen W, Dai F (2020). Genomic signatures and candidate genes of lint yield and fibre quality improvement in Upland cotton in Xinjiang. Plant Biotechnol J.

[57] Thapa R, Tabien RE, Thomson MJ, Septiningsih EM (2020). Genome-wide association mapping to identify genetic loci for cold tolerance and cold recovery during germination in rice. Front Genet.

[58] Chen S, Zhou Y, Chen Y, Gu J (2018). fastp: an ultra-fast all-in-one FASTQ preprocessor. Bioinformatics.

[59] Dobin A, Davis CA, Schlesinger F, Drenkow J, Zaleski C, Jha S, Batut P, Chaisson M, Gingeras TR (2013). STAR: ultrafast universal RNA-seq aligner. Bioinformatics.

[60] Li H, Handsaker B, Wysoker A, Fennell T, Ruan J, Homer N, Marth G, Abecasis G, Durbin R, Genome Project Data Processing S (2009). The sequence alignment/map format and SAMtools. Bioinformatics.

[61] McKenna A, Hanna M, Banks E, Sivachenko A, Cibulskis K, Kernytsky A, Garimella K, Altshuler D, Gabriel S, Daly M, DePristo MA (2010). The genome analysis toolkit: a MapReduce framework for analyzing next-generation DNA sequencing data. Genome Res.

[62] Danecek P, Auton A, Abecasis G, Albers CA, Banks E, DePristo MA, Handsaker RE, Lunter G, Marth GT, Sherry ST (2011). The variant call format and VCFtools. Bioinformatics.

[63] Browning BL, Browning SR (2016). Genotype imputation with millions of reference samples. Am J Hum Genet.

[64] Wang K, Li M, Hakonarson H (2010). ANNOVAR: functional annotation of genetic variants from high-throughput sequencing data. Nucleic Acids Res.

[65] Battle A, Khan Z, Wang SH, Mitrano A, Ford MJ, Pritchard JK, Gilad Y (2015). Genomic variation. Impact of regulatory variation from RNA to protein. Science.

[66] Yang J, Lee SH, Goddard ME, Visscher PM (2011). GCTA: a tool for genome-wide complex trait analysis. Am J Hum Genet.

[67] Purcell S, Neale B, Todd-Brown K, Thomas L, Ferreira MA, Bender D, Maller J, Sklar P, de Bakker PI, Daly MJ, Sham PC (2007). PLINK: a tool set for whole-genome association and population-based linkage analyses. Am J Hum Genet.

[68] Li MX, Yeung JM, Cherny SS, Sham PC (2012). Evaluating the effective numbers of independent tests and significant p-value thresholds in commercial genotyping arrays and public imputation reference datasets. Hum Genet.

[69] Yu G, Wang LG, Han Y, He QY (2012). clusterProfiler: an R package for comparing biological themes among gene clusters. OMICS.

[70] Lu S, Wang J, Chitsaz F, Derbyshire MK, Geer RC, Gonzales NR, Gwadz M, Hurwitz DI, Marchler GH, Song JS (2020). CDD/SPARCLE: the conserved domain database in 2020. Nucleic Acids Res.

[71] Kozomara A, Birgaoanu M, Griffiths-Jones S (2019). miRBase: from microRNA sequences to function. Nucleic Acids Res.

