# Supplementary material for: Mapping intron retention events contributing to complex traits using splice quantitative trait locus
Source: Plant Methods. 2023 Jul 21;19:72. doi: 10.1186/s13007-023-01048-4 (PMC10362629; doi:10.1186/s13007-023-01048-4)
Supplement: Supplementary file 1 — Additional file 1. Additional figures: Figs. S1–S13. Fig. S1. Box plot of Pearson’s correlation coefficient (PCC) of PSI scores among wild cotton accessions. Fig. S2. Box plot of Pearson’s correlation coefficient (PCC) of PSI scores among cultivar cotton accessions. Fig. S3. Filtering of IR events for sQTL mapping. Fig. S4. Pie plot showing the IR events regulated by genetic variation. Fig. S5. Pie charts showing the distribution of PCC values relating IR and gene transcription. Fig. S6. Validation of IR in GhLRRK1. Fig. S7. Validation of IR in GhGC1. Fig. S8. Expression of GhARF3 in different tissues and at different developmental periods, based on FPKM values. Fig. S9. Validation of IR in GhARF3. Fig. S10. Degradome sequencing analysis of the IR. Fig. S11. Expression of GhDCL4 in different tissues and at different developmental periods, based on FPKM values. Fig. S12. Validation of IR in GhDCL4. Fig. S13. Phylogenetic tree representing the relationships among 22 ARF genes of Gossypium hirsutum L. and Arabidopsis thaliana (L.). [file 13007_2023_1048_MOESM1_ESM.pdf]

Mapping Intron Retention Events Contributing to Complex Traits using Splice Quantitative Trait Locus

Siyuan Wang[1], Hongyu Wu[1], Yongyan Zhao[1,2], Luyao Wang[1,2], Xueying Guan[1,2] ,Ting Zhao[1,2*]

1 Zhejiang Provincial Key Laboratory of Crop Genetic Resources, Institute of Crop Science, Plant Precision Breeding Academy, College of Agriculture and Biotechnology, Zhejiang University, Hangzhou, 300058, China.

2 Hainan Institute of Zhejiang University, Building 11, Yonyou Industrial Park, Yazhou Bay Science and Technology City, Yazhou District, Sanya, Hainan, 572025, China

* Corresponding author. Correspondence and requests for materials should be addressed to Ting Zhao (tingzhao@zju.edu.cn)

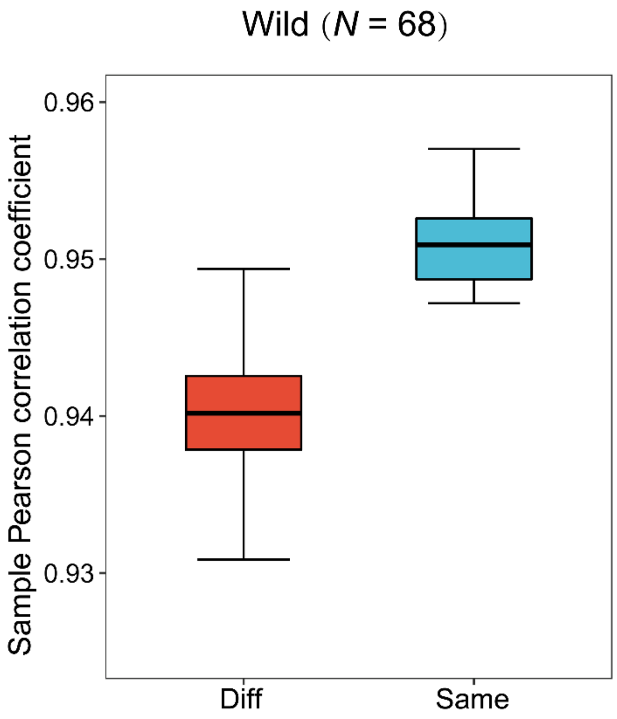

Fig. S1 Box plot of Pearson's correlation coefficient (PCC) of PSI scores among wild cotton accessions. "Same" and "diff" denote whether the two samples are biological replicates. Boxes span the first to third quartiles and center lines indicate the second quartile (median).

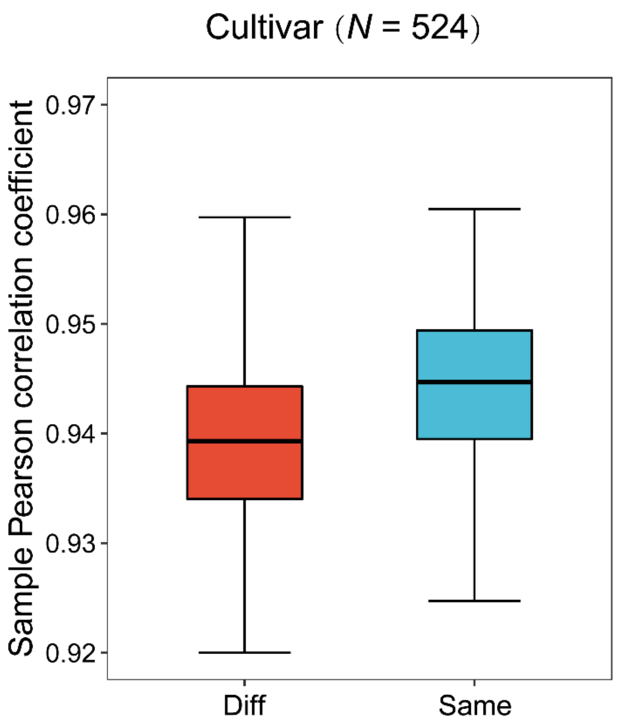

Fig. S2 Box plot of Pearson's correlation coefficient (PCC) of PSI scores among cultivar cotton accessions. "Same" and "diff" denote whether the two samples are biological replicates. Boxes span the first to third quartiles and center lines indicate the second quartile (median).

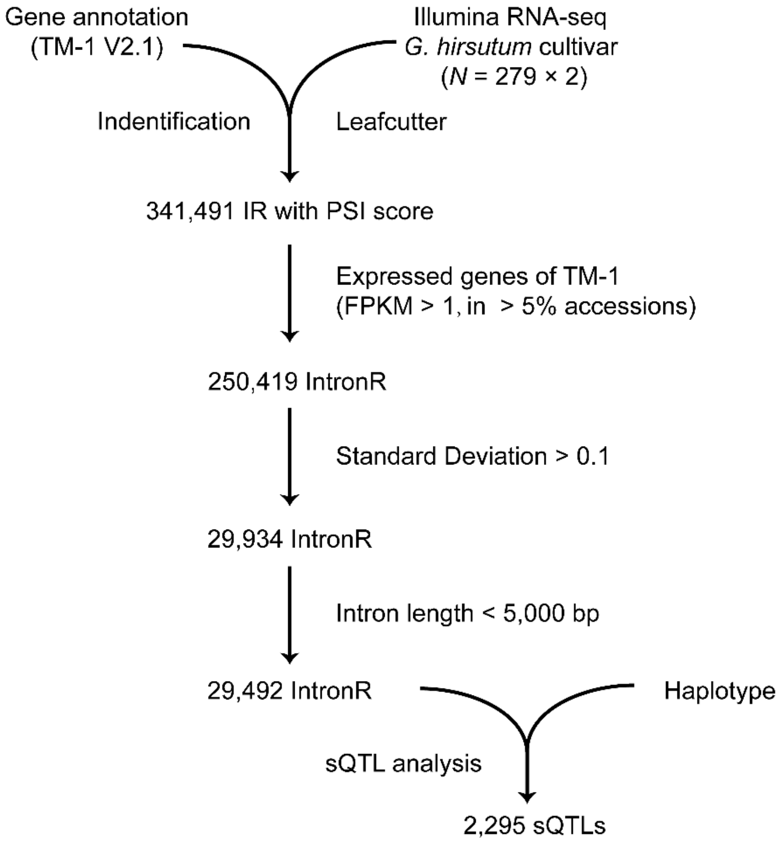

Fig. S3 Work flow of filtering IR events for sQTL mapping. First, IR events in 1-DPA ovule RNA-seq were identified by comparison to the reference genome (TM-1 v2.1) using Leafcutter software. Then, the 341,491 events identified were filtered according to the following criteria: (i) high expression (FPKM ≥ 1 at the gene level for 95% of germplasms in the population); (ii) high variation in PSI (for each IR event, standard deviation > 0.1 in the population); and (iii) intron length < 5,000 bp. A total of 29,492 IR events were retained and used for genome-wide association analysis with 1,186,673 biallelic SNPs (MAF > 0.05, missing rate < 20%), which yielded 2,295 sQTLs.

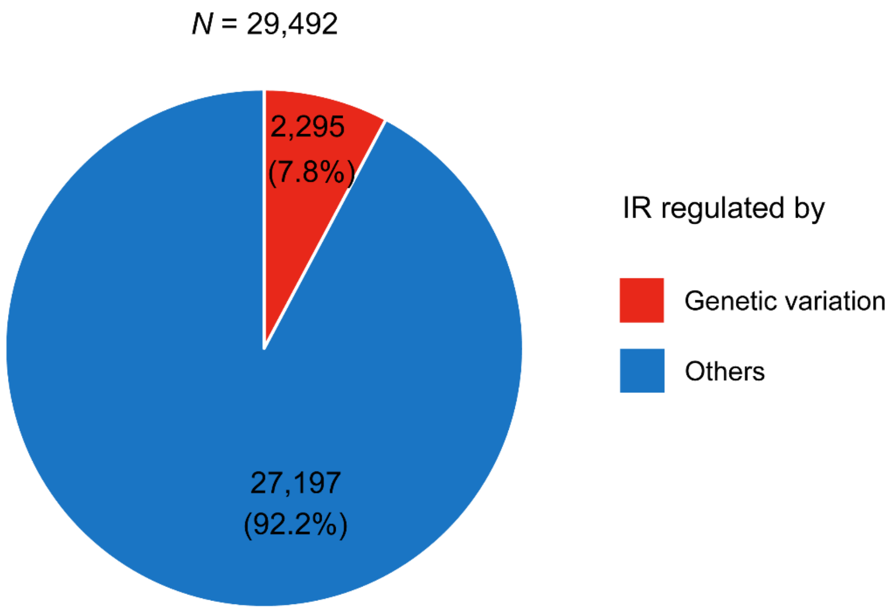

Fig. S4 Pie plot showing the IR events regulated by genetic variation.

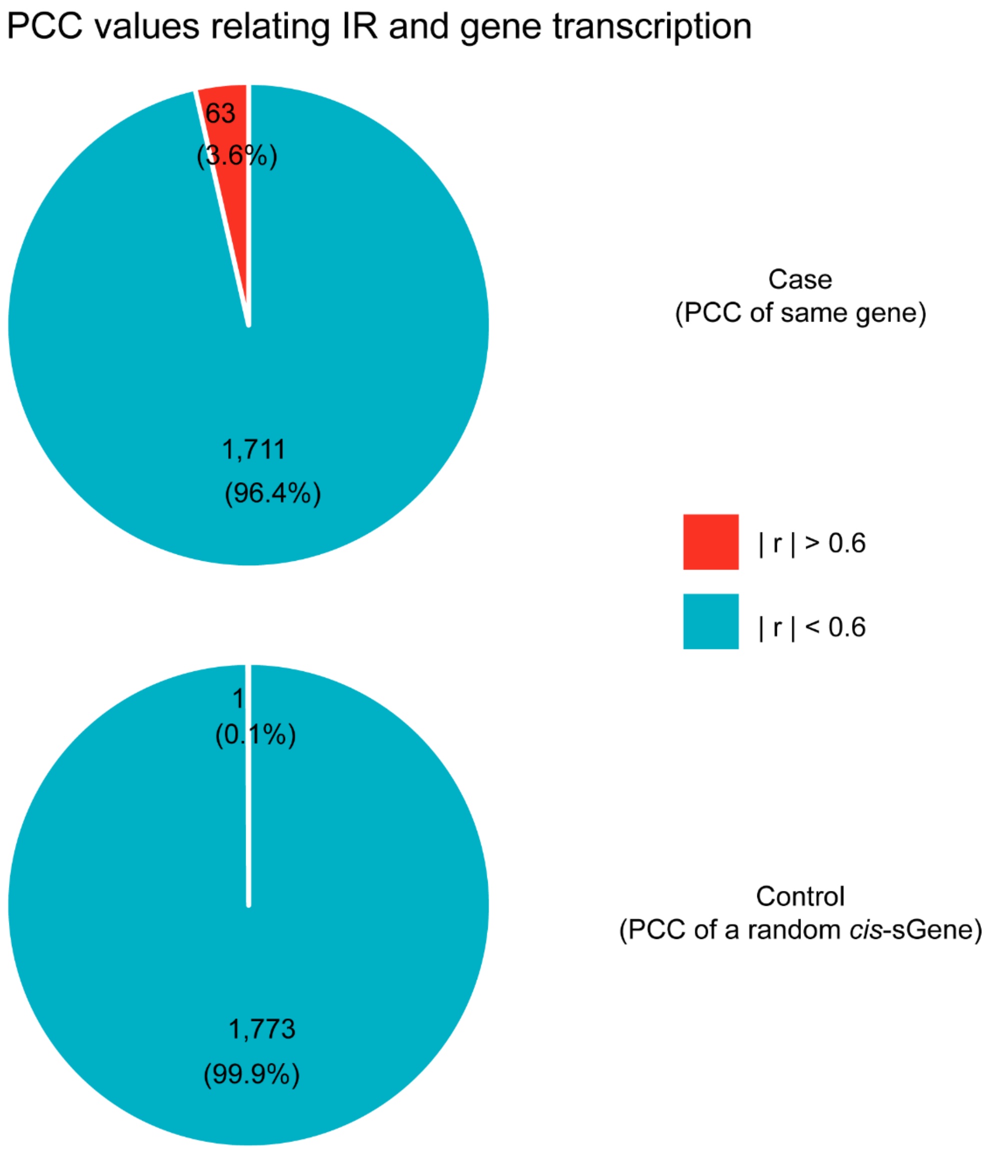

Fig. S5 Pie charts showing the distribution of PCC values relating IR and gene transcription. Case: PCC was calculated for IR and expression of the same gene. Control: PCC was calculated for IR and the expression of a randomly selected *cis*-sQTL gene.

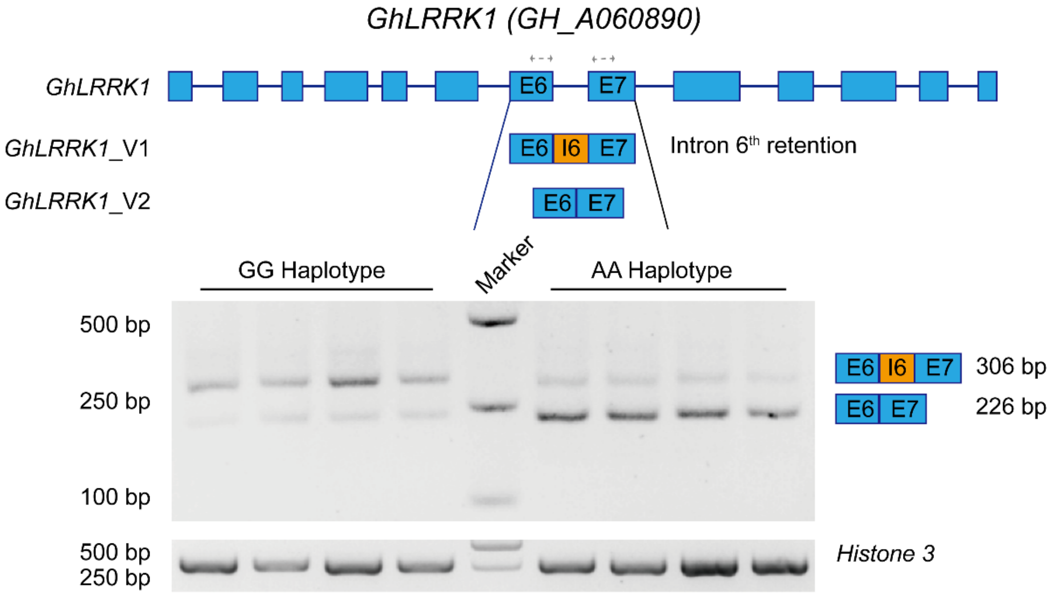

Fig. S6 Validation of IR in *GhLRRK1*. Gene models depict *GhLRRK1* isoforms from TM-1 v2.1 and show retention of the 6th intron. The gel image shows RT-PCR amplification of cDNA from 1-DPA ovule RNA of random accession samples representing each of the two haplotypes (GG and AA) at sSNP A06:23513733. *Histone3* was used as a control. The positions of the primers used for RT-qPCR are indicated on the gene model by dotted lines with arrows.

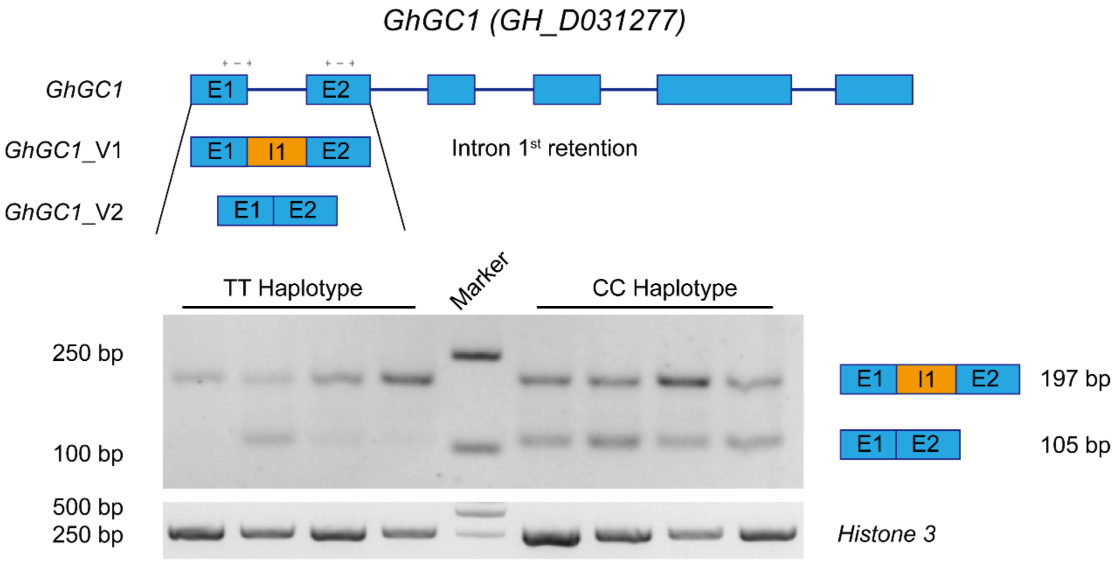

Fig. S7 Validation of IR in *GhGC1*. Gene models depict *GhGC1* isoforms from TM-1 v2.1 and show retention of the 1st intron. The gel image shows RT-PCR amplification of cDNA from 1-DPA ovule RNA of random accession samples representing each of the two haplotypes (TT and CC) at sSNP D03:43244243. *Histone3* was used as a control. The positions of the primers used for RT-qPCR are indicated on the gene model by dotted lines with arrows.

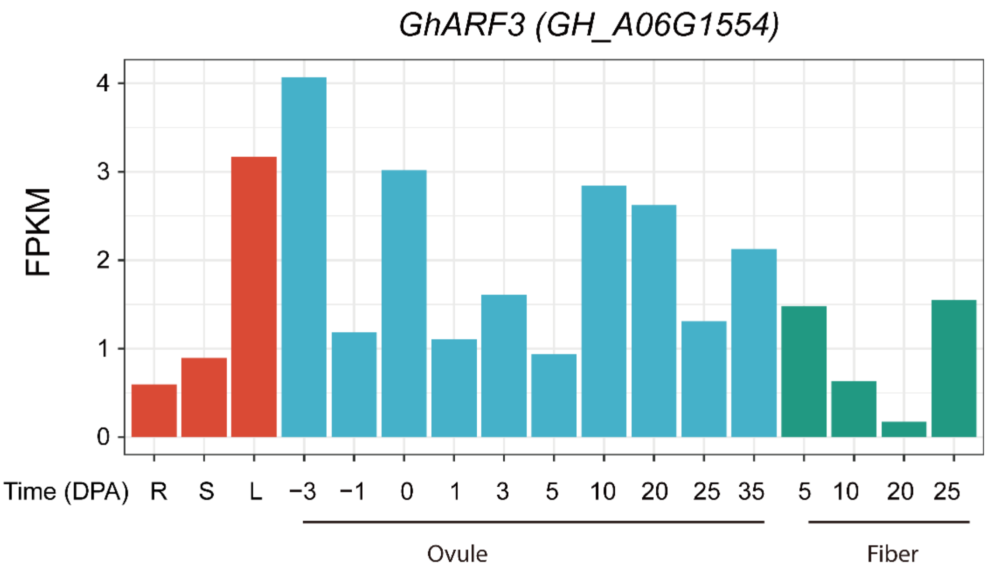

Fig. S8 Expression of *GhARF3* in different tissues and at different developmental periods, based on FPKM values. The x-axis lists the different tissues, including root, stem, leaf, ovule, and fiber. Ovule tissue was collected 3 and 1 days before flowering, the day of flowering, and 1 to 35 days after flowering. Fiber tissue was collected 5 to 25 days after flowering.

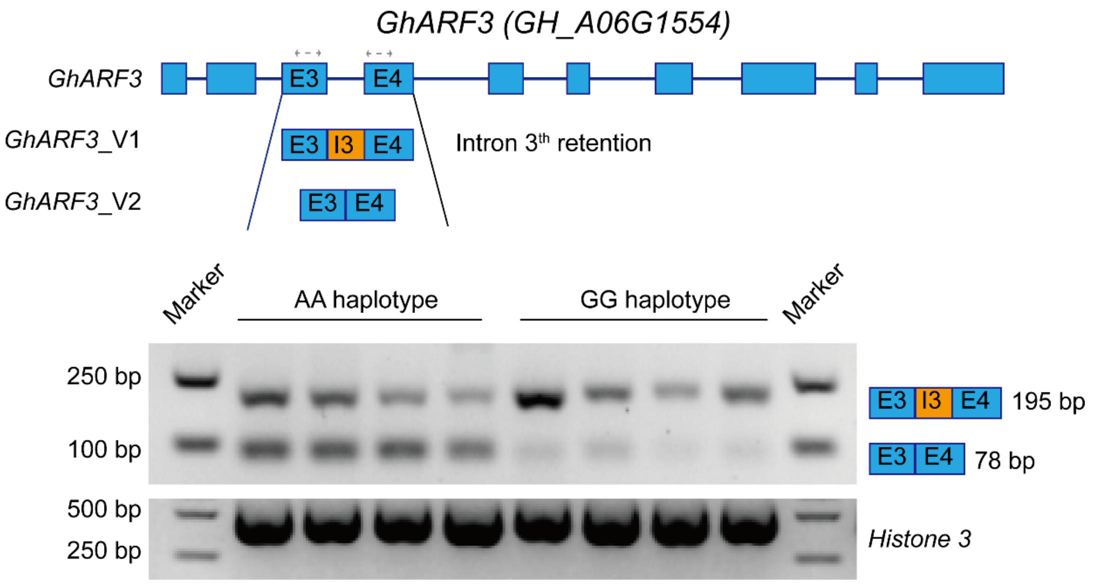

Fig. S9 Validation of IR in *GhARF3*. Gene models depict *GhARF3* isoforms from TM-1 v2.1 and show retention of the 3th intron. The gel image shows RT-PCR amplification of cDNA from 1-DPA ovule RNA of random accession samples representing each of the two haplotypes (AA and GG) at sSNP A06:105487159. *Histone3* was used as a control. The positions of the primers used for RT-qPCR are indicated on the gene model by dotted lines with arrows.

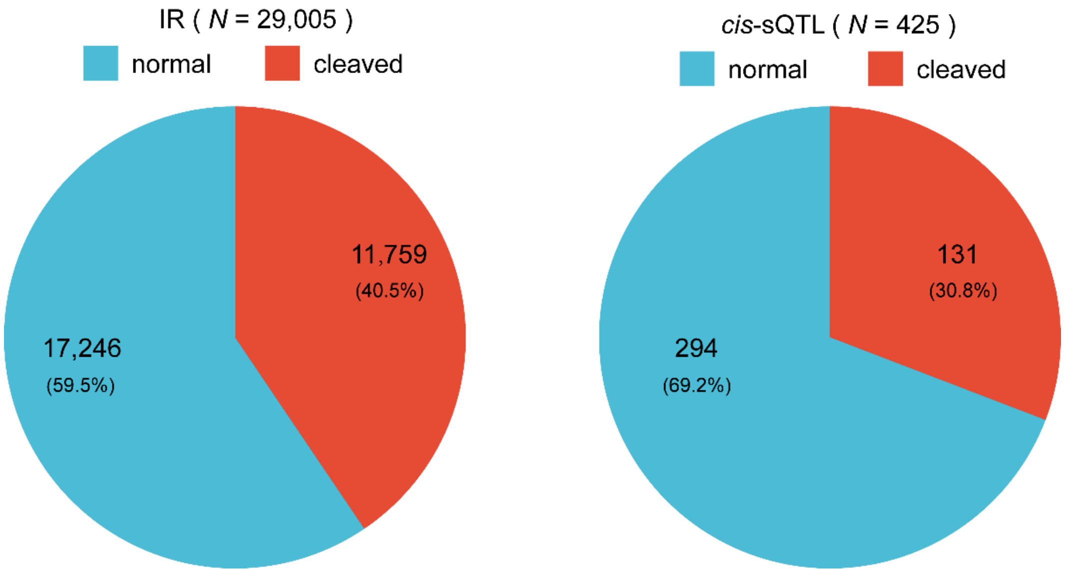

Fig. S10 Degradome sequencing analysis of the IR. Pie charts showing the distribution of the cleavage of candidate IR (left) and *cis*-sQTL (right). Cleaved: the locus will be degraded, normal: no degradation. The degradome data from Cao et al. (2020).

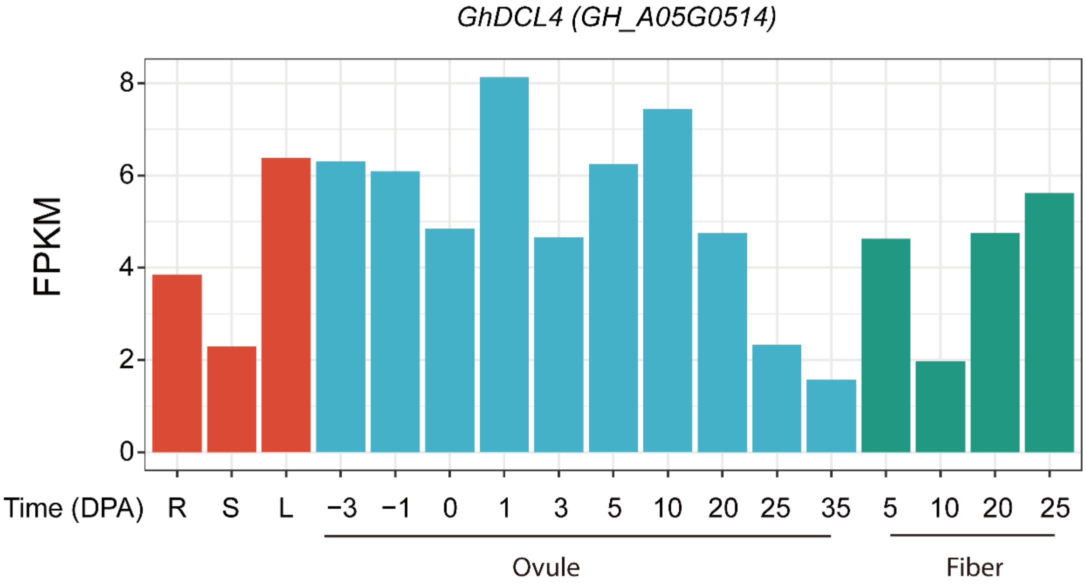

Fig. S11 Expression of *GhDCL4* in different tissues and at different developmental periods, based on FPKM values. The x-axis lists the different tissues, including root, stem, leaf, ovule, and fiber. Ovule tissue was collected 3 and 1 days before flowering, the day of flowering, and 1 to 35 days after flowering. Fiber tissue was collected 5 to 25 days after flowering.

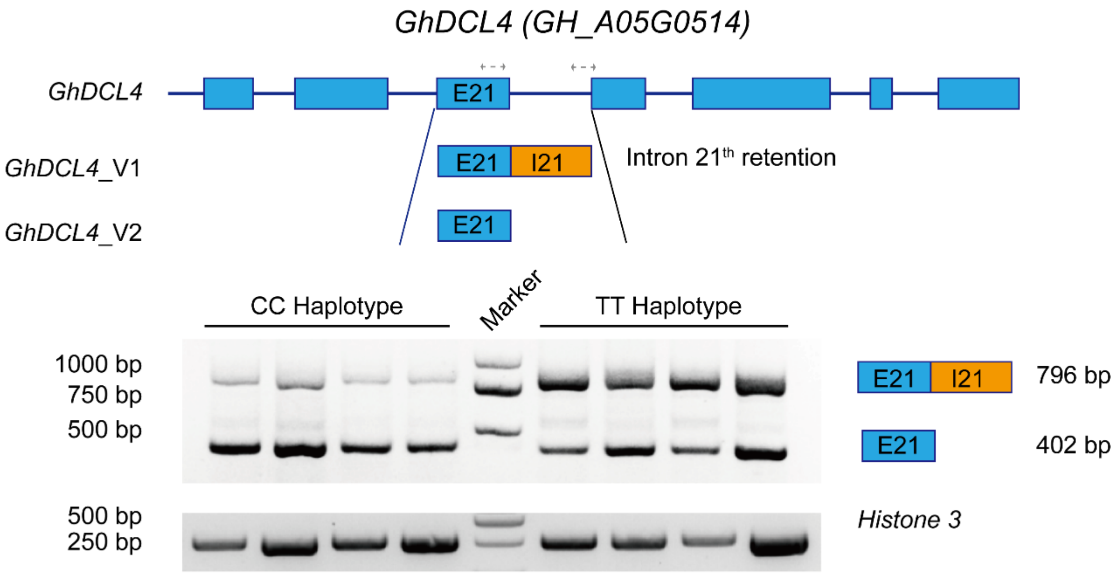

Fig. S12 Validation of IR in *GhDCL4*. Gene models depict *GhDCL4* isoforms from TM-1 v2.1 and show retention of the 21th intron. The gel image shows RT-PCR amplification of cDNA from 1-DPA ovule RNA of random accession samples representing each of the two haplotypes (CC and TT) at sSNP A05:4938322. *Histone3* was used as a control. The positions of the primers used for RT-qPCR are indicated on the gene model by dotted lines with arrows.

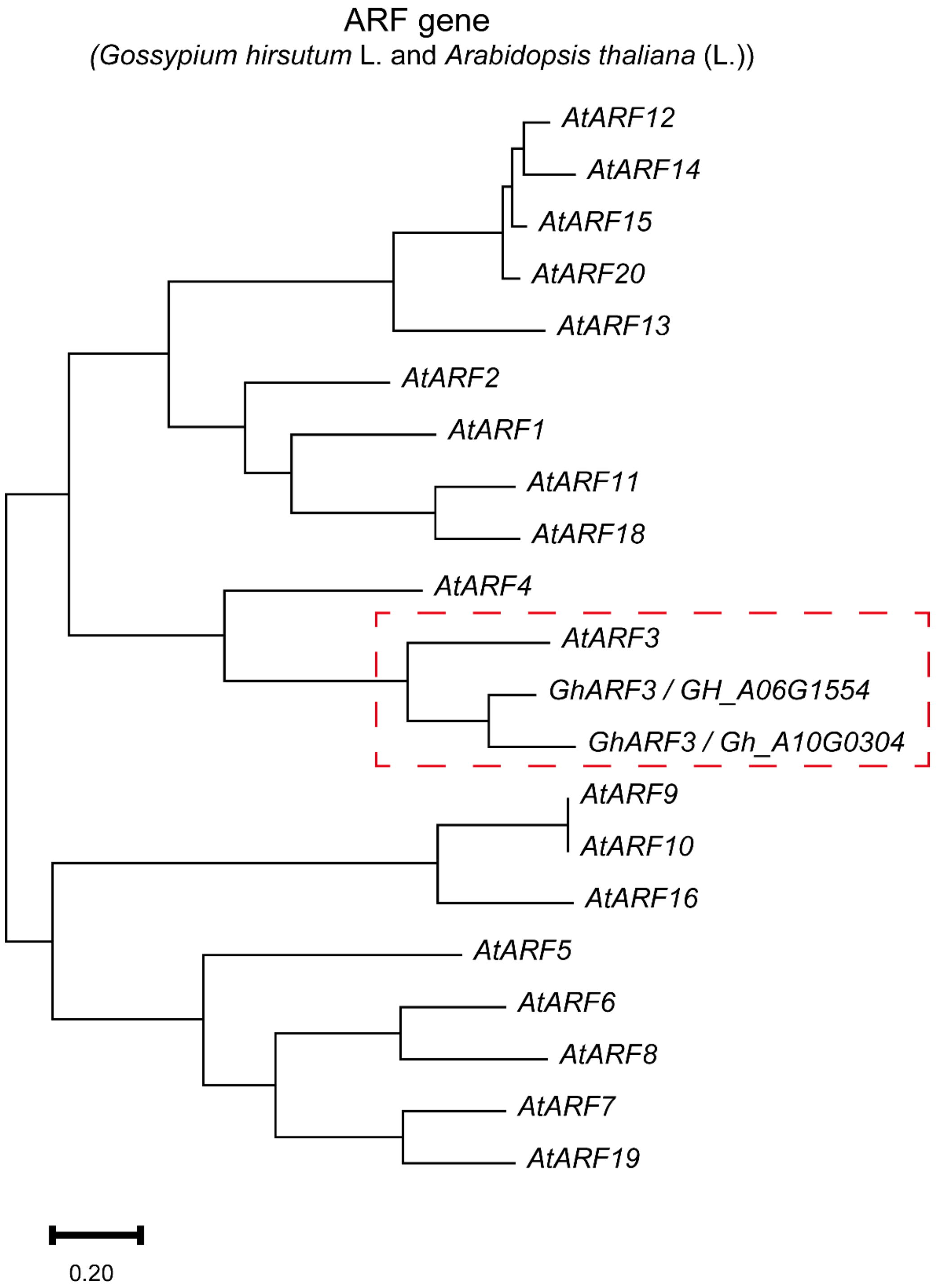

Fig. S13 Phylogenetic tree representing the relationships among 22 ARF genes of *Gossypium hirsutum* L. and *Arabidopsis thaliana* (L.). Tree was constructed with 1000 bootstrap replicates by the maximum likelihood (ML) method. The red box highlights the orthology of *GhARF3* (*GH_D06G1524*) to *Gh_A10G0304* and *AtARF3*.
